# An AI-supported E-mentoring model to develop EFL pre-service teachers’ self-efficacy and emotional intelligence

**DOI:** 10.3389/fpsyg.2026.1853510

**Published:** 2026-09-07

**Authors:** Israa Ismael, Xin Luo, Sen Li

**Affiliations:** 1 Department of Curriculum and Instruction, School of Education, Shaanxi Normal University, Xi’an, China; 2 Department of Early Childhood Education, School of Education, Shaanxi Normal University, Xi’an, China

**Keywords:** AI-supported mentoring, EFL pre-service teachers, emotional intelligence, practicum, teacher self-efficacy

## Abstract

**Introduction:**

This study investigated the effectiveness of an AI-supported e-mentoring model in enhancing self-efficacy and emotional intelligence among EFL pre-service teachers within the Egyptian practicum context.

**Methods:**

Using a mixed-methods quasi-experimental design, 50 participants were assigned to an experimental group, which received structured mentoring, collaborative digital platform support (Facebook, Padlet, Nearpod, Google Classroom), and AI-driven feedback via Gemini, and a control group, which followed conventional practicum procedures.

**Results:**

Quantitative results revealed substantial improvements in the experimental group’s self-efficacy and emotional intelligence compared to the control group, while qualitative data indicated that participants shifted toward more student-centered, reflective, and adaptive teaching practices.

**Discussion:**

The findings highlight the potential of integrating AI tools with structured mentoring and collaborative digital environments to create a continuous cycle of practice, feedback, and reflection. Implications for teacher education emphasize scalable, context-sensitive professional development that addresses both pedagogical and emotional competencies, particularly in under-resourced settings. Study limitations and directions for future research are also discussed.

## 1 Introduction

The effectiveness of any educational system depends largely on the quality of its teachers, making teacher preparation a central priority in educational reform worldwide, with emphasis on both pedagogical knowledge and professional competencies (Villocino and Villocino, 2025). For EFL pre-service teachers, preparation is particularly demanding, as they must integrate linguistic competence, instructional skills, and interpersonal abilities in increasingly complex, technology-mediated environments (Abildinova et al., 2024; Tondeur et al., 2025).

Among the key competencies required for effective teaching are self-efficacy and emotional intelligence. Rooted in Bandura’s Social Cognitive Theory, self-efficacy refers to individuals’ beliefs in their capabilities to successfully perform specific tasks under varying conditions (Bandura, 1997). In educational contexts, teachers with high self-efficacy demonstrate greater persistence, instructional flexibility, and classroom management effectiveness, which in turn positively influence student outcomes (Eren et al., 2025; Li, 2023). However, research indicates that many pre-service teachers enter practicum experiences with underdeveloped self-efficacy, often due to limited exposure to authentic teaching situations and insufficient feedback mechanisms (Masood et al., 2022; Kozikoglu and Senemoglu, 2021).

Complementing this cognitive dimension, emotional intelligence (EI) plays a critical role in teaching effectiveness. Emotional intelligence encompasses the ability to perceive, understand, regulate, and utilize emotions in oneself and others (Goleman, 1996; Mayer et al., 1999). In EFL contexts, EI is vital for fostering supportive learning environments, managing classroom challenges, and building positive student relationships (Chen et al., 2024; Paschal et al., 2024). However, many teacher education programs inadequately address EI, leaving pre-service teachers underprepared for emotional demands (Masood et al., 2022).

These challenges are particularly evident during the Egyptian practicum, where many pre-service teachers experience stress anxiety, and uncertainty while navigating classroom management, instructional delivery, and professional expectations (Alamri, 2018; Asrida et al., 2025). Additionally, mentoring systems tend to be inconsistent and primarily focused on evaluation rather than continuous developmental guidance (Abdallah, 2025; Mekheimer, 2025). As a result, many EFL pre-service teachers report feelings of isolation and insufficient preparedness, highlighting the need for more structured and supportive mentoring approaches.

Despite the well-documented importance of mentoring in teacher education, existing mentoring practices in EFL teacher preparation programs remain largely traditional, inconsistent, and limited in scope. Research indicates that practicum supervision is often characterized by irregular feedback, emphasis on evaluation rather than developmental support, and limited opportunities for sustained reflection and guided practice (Abdallah, 2025; Mekheimer, 2025). In many cases, mentoring is dependent on individual supervisors’ availability and expertise, resulting in variability in the quality of support provided to pre-service teachers (Kozikoglu and Senemoglu, 2021). Consequently, many pre-service EFL teachers experience difficulty bridging the gap between theoretical knowledge and real classroom practice, which negatively affects both their teaching self-efficacy and emotional preparedness.

To address these limitations, e-mentoring and digitally supported peer mentoring models have been introduced as alternative approaches that extend mentoring beyond physical and temporal constraints. These models enable continuous interaction, collaborative reflection, and shared professional learning (Gunawardena, 2023; Zhang et al., 2025). However, despite their advantages, e-mentoring systems still rely heavily on human mentors and remain constrained by variability in feedback quality, scalability, and workload demands.

In parallel, artificial intelligence (AI) has emerged as a promising tool in education, offering capabilities such as automated feedback, adaptive scaffolding, and personalized instructional support. However, in teacher education, AI is still largely used as an isolated instructional tool rather than being systematically embedded within mentoring frameworks. More importantly, its potential role in supporting both cognitive (self-efficacy) and affective (emotional intelligence) dimensions of teacher development remains underexplored.

A critical gap therefore exists at the intersection of mentoring, AI integration, and teacher professional development. Specifically, there is a lack of empirical research examining AI-supported e-mentoring as an integrated system that combines human guidance with AI-driven adaptive feedback to simultaneously enhance both self-efficacy and emotional intelligence. This gap is particularly significant in EFL teacher education contexts in developing countries, where practicum demands are high and mentoring resources are limited.

To address this gap, the present study designs and implements an AI-supported e-mentoring model that integrates human mentoring, collaborative digital platforms, and AI-driven tools to provide adaptive instructional and emotional support. The model aims to enhance both self-efficacy and emotional intelligence among EFL pre-service teachers during their practicum experience.

## 2 Literature review

### 2.1 Self-efficacy in teacher education

Self-efficacy, a central construct in teacher education, refers to teachers’ beliefs in their ability to organize and execute instructional practices that achieve desired student outcomes (Bandura, 1997). Grounded in Social Cognitive Theory, self-efficacy develops through four primary sources: mastery experiences, vicarious experiences, social persuasion, and emotional states (Bandura, 1977; Lerner and Hess, 1999). Among these, mastery experiences are consistently identified as the strongest predictor of sustained efficacy development in pre-service teachers.

It is important to situate self-efficacy within the broader international discussion of teacher professional development and teacher competence, rather than treating it as a stand-alone predictor of teaching quality. Influential models of effective teacher professional development identify self-efficacy as one of several interrelated outcomes, alongside content knowledge, pedagogical skill, and instructional practice, that emerge from sustained, content-focused, and collaborative learning experiences (Desimone, 2009; Guskey, 2002). Similarly, internationally recognized teacher competence frameworks conceptualize effective teaching as a multidimensional construct encompassing subject-matter knowledge, pedagogical and didactic skill, classroom management, collaborative and reflective capacity, and socio-emotional competence, of which self-efficacy beliefs represent only one component (Darling-Hammond et al., 2017). Positioning self-efficacy within this wider architecture clarifies that, while it is a necessary condition for effective teaching, it is not sufficient on its own, and its development should be understood as embedded within, and shaped by, the broader professional and pedagogical competencies that teacher preparation programs aim to cultivate.

Empirical research across diverse contexts has consistently demonstrated the importance of teacher self-efficacy in shaping instructional quality and classroom effectiveness. In large-scale studies in the United States and Europe, higher self-efficacy has been associated with increased classroom management effectiveness, greater instructional flexibility, and stronger student engagement outcomes (Tschannen-Moran and Hoy, 2001; Klassen and Tze, 2014). Similarly, studies in Asian EFL contexts have shown that teachers with stronger self-efficacy are more likely to implement communicative language teaching strategies and sustain learner-centered instruction despite contextual constraints (Choi and Lee, 2018; Songhori and Mehr, 2025).

Intervention-based studies further confirm that self-efficacy is not static but can be developed through structured professional experiences. For example, practicum-based research has shown that guided reflection, mentoring support, and repeated teaching practice significantly enhance pre-service teachers’ efficacy beliefs (Chesnut and Burley, 2015; Zee and Koomen, 2016). In digitally supported environments, collaborative reflection and online peer feedback have also been found to strengthen teacher confidence by providing continuous social persuasion and opportunities for observational learning (Trust and Whalen, 2020; Li, 2023).

However, despite these advances, research consistently highlights that self-efficacy development in teacher education remains uneven and highly context-dependent. In many practicum settings, including both Western and non-Western contexts, pre-service teachers report a lack of structured opportunities for mastery experiences and insufficient formative feedback (Flores, 2015; Klassen and Tze, 2014). These limitations are particularly pronounced in EFL teacher education programs in developing contexts, where institutional constraints often restrict classroom exposure and reduce opportunities for iterative practice.

In the Middle East and North African region, similar patterns have been documented. Studies indicate that pre-service EFL teachers frequently experience a gap between theoretical preparation and real classroom demands, leading to uncertainty in instructional decision-making and classroom management (El-Henawy, 2025; Abdallah, 2025). Feedback is often summative rather than developmental, limiting its impact on efficacy growth, while mentoring practices vary widely in quality and consistency.

Additionally, recent research emphasizes that teacher education programs often address self-efficacy implicitly rather than through structured developmental frameworks. Without intentional scaffolding, reflective cycles, and sustained mentoring support, self-efficacy tends to remain fragile and context-dependent rather than stable and transferable (Kite and Durksen, 2026; Shi et al., 2025a, 2025b).

Overall, the literature suggests that while self-efficacy is widely recognized as a critical, though not exclusive, determinant of teacher effectiveness, its development among pre-service EFL teachers remains constrained by limited authentic teaching experiences, inconsistent mentoring support, and insufficient structured reflection. These persistent challenges highlight the need for integrated, sustained, and adaptive approaches that combine real teaching practice, continuous feedback, and reflective support systems within a broader competence-based view of teacher development.

### 2.2 Emotional intelligence in EFL teaching

Emotional intelligence (EI) refers to the ability to perceive, understand, regulate, and effectively utilize emotions in oneself and others (Mayer et al., 1999; Goleman, 1998). Within teacher education, EI is linked to reduced stress, burnout, and enhanced classroom management, instructional effectiveness, and professional resilience (Geraci et al., 2026; Sasere and Matashu, 2025). It should be noted that EI is not a single, uniformly agreed-upon construct, but is theorized and measured in at least two distinct ways: the ability model, which treats EI as a form of intelligence involving the cognitive processing of emotional information and is assessed through performance-based tasks (Mayer et al., 1999), and the trait model, which conceptualizes EI as a constellation of self-perceived emotional dispositions located at the lower levels of the personality hierarchy and is assessed through self-report questionnaires such as the TEIQue (Petrides et al., 2007). These two models are not interchangeable, and findings from one cannot be generalized to the other; the present study adopts the trait EI perspective, given its use of the TEIQue as the measurement instrument.

The conceptual status of EI, particularly trait EI, has also been the subject of ongoing scholarly debate. Locke (2005) argued that EI, as commonly operationalized, does not meet the criteria for a coherent scientific construct, contending that what is labeled “emotional intelligence” largely reflects a loosely bundled set of pre-existing personality traits and social skills rather than a distinct form of intelligence. Consistent with this critique, empirical studies have repeatedly shown that trait EI correlates substantially with established Big Five personality dimensions, particularly low neuroticism (emotional stability), raising questions about its discriminant validity and whether it captures variance beyond well-established personality constructs (Alegre et al., 2019; Petrides et al., 2007). This overlap has led some researchers to characterize trait EI as a proxy for broader personality dispositions rather than an independent psychological construct in its own right.

Despite these conceptual and psychometric concerns, trait EI models such as Petrides’ (2009) framework have demonstrated adequate internal consistency and have shown incremental predictive validity for outcomes such as well-being, stress regulation, and coping beyond that accounted for by personality alone (Petrides et al., 2007). Within teacher education specifically, trait EI has been used as a practical, self-report indicator of teachers’ perceived emotional competencies, including emotional awareness, regulation, and interpersonal functioning, which are directly relevant to the demands of classroom teaching, even though it does not resolve the broader theoretical debate about whether EI constitutes a distinct form of intelligence. The present study therefore treats trait EI, as measured by the TEIQue, as an indicator of self-perceived emotional functioning relevant to teaching practice, rather than as an uncontested measure of an underlying emotional intelligence.

In EFL classrooms, where communication is central, teachers must manage affective dynamics such as anxiety, hesitation, and intercultural differences (Lasekan et al., 2025; Zhi et al., 2023; Li and Zhang, 2024). Thus, high EI enables teachers to create supportive classroom climates, foster empathy, and facilitate meaningful interaction, all of which are essential for communicative language teaching (Awwad, 2022). Moreover, emotionally intelligent teachers are better equipped to regulate their own stress, adapt to diverse learner needs, and maintain positive interpersonal relationships, thereby enhancing both teaching effectiveness and student engagement (Yang and Duan, 2023).

On the other hand, EI is closely intertwined with SE. Teachers who can regulate their emotions, manage stress, and respond constructively to classroom challenges are more likely to develop stronger beliefs in their instructional capabilities (Wang and Wang, 2022). In this sense, EI functions as a foundational mechanism that supports the development of self-efficacy by enabling successful teaching experiences and sustained effort in the face of difficulties (Kyriazopoulou et al., 2025). Conversely, limited emotional regulation may undermine SE, as pre-service teachers may perceive themselves as less capable of handling emotionally demanding situations. Empirical studies across diverse contexts have shown that higher EI, particularly intrapersonal and interpersonal competencies, predicts stronger self-efficacy across classroom management, student engagement, and instructional practices (Barari and Barari, 2015; Yuan et al., 2025).

Empirical evidence indicates that intrapersonal and interpersonal EI competencies predict stronger self-efficacy across classroom management, student engagement, and instructional practices (Noor and Gamilina, 2026; Yuan et al., 2025). However, few studies address integrated EI and SE interventions for EFL pre-service teachers in Egypt, highlighting a critical gap in teacher preparation programs. Moreover, EI remains under-theorized in teacher education within the Egyptian context. Many pre-service teachers enter classrooms without structured opportunities to develop their emotional competencies, leaving them underprepared for the relational and affective demands of teaching (Braun and Hooper, 2024; Kızıldağ and Kırmızı, 2025). This reflects a critical limitation in current teacher preparation programs, which tend to overlook the systematic and integrated development of emotional intelligence alongside self-efficacy, particularly in emotionally demanding EFL classrooms.

### 2.3 E-mentoring and AI-enhanced mentoring in teacher education

E-mentoring extends traditional mentoring by providing flexible, accessible developmental support through digital platforms (Allen et al., 2004; Villani, 2009). Within teacher preparation, E-mentoring extends the mentor’s role beyond direct instruction to include coaching, scaffolding reflection, and supporting the development of professional identity (Bell, 2024; Erdoğan et al., 2021). Importantly, e-mentoring fosters communities of practice in which pre-service teachers can share experiences, discuss challenges, and engage in reflective dialogue (Hobson et al., 2009; Kaçar and Baltacı, 2023). These interactions not only promote professional growth but also provide essential emotional support during the initial practicum years, helping novice teachers develop confidence, strengthen their professional identity, and navigate the stress and isolation commonly experienced in real classroom contexts (Burger et al., 2021; Elemdag and Erdem, 2017).

However, mentoring relationships are often shaped by hierarchical structures that position mentors as evaluators rather than developmental supporters, leading to what has been described as “judge mentoring,” where feedback becomes directive and prescriptive rather than reflective and dialogic (Ayaz et al., 2026). Even within e-mentoring environments, feedback tends to be generalized, delayed, or inconsistent, limiting its effectiveness in supporting real-time instructional improvement (Jan and Mehboob, 2022). A further critical limitation lies in the lack of personalization and continuity: human mentors may struggle to provide timely, sustained feedback, which constrains the capacity for ongoing, adaptive guidance (Bell, 2024). As a result, existing mentoring approaches fall short of providing timely, adaptive, and individualized support throughout the practicum process.

In this context, advances in artificial intelligence (AI) offer new possibilities for addressing these limitations. AI-driven systems can provide immediate, adaptive feedback based on pre-service teachers’ inputs, enabling timely intervention during lesson planning and reflection (He et al., 2025). Unlike traditional mentoring, which often relies on fragmented observations, AI can continuously analyze patterns across multiple data points, including reflective entries, instructional artifacts, and interaction logs (Nygren et al., 2025). This allows for the identification of recurring challenges related to instructional practices, emotional responses, and engagement levels. In turn, AI can generate targeted suggestions, recommend relevant resources, and support reflective practice in a more structured and responsive manner (Miy, 2026). When integrated into e-mentoring frameworks, AI can further enhance reflective processes, facilitate adaptive support, and maintain a continuous record of professional growth, enabling pre-service teachers to monitor improvements in both self-efficacy and emotional regulation with greater clarity and evidence (Samuels et al., 2025).

Despite growing interest, research has largely treated e-mentoring and AI separately. Few studies systematically examine dual development of SE and EI among EFL teachers, particularly in under-resourced contexts like Egypt, underscoring the need for integrated AI-supported e-mentoring models.

### 2.4 Teacher preparation in the Egyptian EFL context

In Egypt, teacher preparation typically follows a sequential model that prioritizes theoretical coursework at the university level, followed by a practicum phase in real school settings (Mekheimer, 2025). While this structure is intended to develop professional competence, it often exposes a persistent theory-practice gap. Pre-service EFL teachers frequently report that their academic preparation does not adequately equip them for the complexities of real classrooms, particularly in areas such as classroom management, instructional decision-making, and communicative language teaching (Masood et al., 2022). Empirical studies further indicate that teaching methods courses are often insufficiently aligned with practicum demands, leaving student-teachers struggling to transfer theoretical knowledge into practice (Abdallah, 2025; El-Henawy, 2025; El-Kerdany, 2013). This disconnect is further compounded by contextual constraints in schools, including overcrowded classrooms, exam-oriented practices, and limited instructional resources, all of which restrict the implementation of student-centered and communicative approaches (Rezk, 2009).

Moreover, pre-service teachers often enter schools as “guests” rather than active professionals (Moustafa et al., 2026). Some are given full teaching responsibilities, but many are sidelined or assigned to substitute lessons outside their subject area (Elghotmy, 2012). Combined with challenges like limited language proficiency and fear of negative evaluation from supervisors or students, these factors can lower self-efficacy and hinder emotional development (R’boul, 2025). Thus, the Egyptian practicum context reflects a critical gap in structured, supportive, and development-oriented training, highlighting the need for more integrated models that address both the pedagogical and emotional dimensions of pre-service teacher development.

Building on this need, recent research has begun to explore the potential of AI-supported e-mentoring in enhancing learning and professional development. Evidence suggests that AI-integrated mentoring can support self-regulated learning, engagement, and reflective practice through adaptive feedback and personalized guidance (Hussain et al., 2025; Kellam et al., 2025).

However, most studies emphasized self-regulated learning, engagement, or performance outcomes, with limited attention to core teacher-related constructs such as self-efficacy and emotional intelligence. Moreover, while some studies incorporate AI within mentoring contexts, they often do so at a functional or exploratory level, without developing structured, theory-driven models that systematically integrate adaptive AI support within mentoring processes. This limitation is further compounded by the scarcity of research situated in EFL teacher education, particularly within under-resourced contexts such as Egypt, where the need for continuous, personalized, and emotionally responsive support is most critical. This gap underscores the need for more comprehensive models that address both pedagogical competence and socio-emotional development within context-specific constraints.

Therefore, this current study seeks to address the previously mentioned challenges and gaps through the following research questions:

- How do EFL pre-service teachers experience and describe changes in their self-efficacy and emotional intelligence throughout their participation in the AI-supported e-mentoring model?
- To what extent does the model improve EFL pre-service teachers’ self-efficacy?
- To what extent does the model improve EFL pre-service teachers’ emotional intelligence?

## 3 A conceptual model for AI-supported E-mentoring in EFL teacher education

### 3.1 Theoretical foundations

This study is grounded in the broader international literature on teacher professional development (TPD) and teacher competence, which views effective teaching as the outcome of sustained, content-focused, and collaborative learning experiences that shape multiple, interrelated aspects of a teacher’s growth, including knowledge, skill, self-efficacy, and socio-emotional functioning (Darling-Hammond et al., 2017; Desimone, 2009; Guskey, 2002). Within this wider architecture, self-efficacy and emotional intelligence are not treated as the whole of teacher professionalism, but as two theoretically grounded and measurable dimensions of it, selected here for focused investigation.

To account for the development of self-efficacy, the model draws on Bandura’s (1997) Social Cognitive Theory, which identifies mastery experiences, vicarious experiences, social persuasion, and emotional states as the primary sources of efficacy beliefs. Structured mentoring and continuous feedback are the two mechanisms through which the model puts these sources into practice, allowing pre-service teachers to strengthen their instructional confidence through repeated, reflective engagement with real classroom experience.

Feedback is understood following Hattie and Timperley’s (2007) model, as information given to a learner about their performance in relation to a goal, organized around three questions, Where am I going? (feed-up), How am I going? (feed-back), and Where to next? (feed-forward), operating at the task, process, self-regulation, and self-levels, with task- and process-level feedback showing the strongest link to skill development. The present model reflects this distinction directly: AI-generated feedback mainly addresses the task and process levels through immediate, strategy-specific suggestions, while human mentor feedback works more at the self-regulation and self-levels, supporting reflection, emotional processing, and the development of professional identity.

Alongside self-efficacy, the model addresses the affective and interpersonal side of teaching through emotional intelligence, adopting the trait EI perspective (Petrides et al., 2007) rather than the ability-based model of EI as a distinct form of intelligence (Mayer et al., 1999). This choice matches the study’s use of the TEIQue and comes with an explicit caveat: trait EI remains a debated construct, with documented overlap with established personality dimensions (Alegre et al., 2019; Locke, 2005). Accordingly, EI is treated here as a practically useful, self-reported indicator of emotional functioning relevant to teaching, not as a settled construct with the same theoretical standing as self-efficacy.

Taken together, self-efficacy and emotional intelligence are treated as distinct but related constructs, both developed through guided practice, reflection, and adaptive support, and both representing key dimensions of the broader teacher competence this mentoring model aims to build.

### 3.2 Model overview

The AI-supported e-mentoring model is a dynamic, iterative system integrating human mentoring with AI-driven support to enhance both pedagogical and emotional development, operating through continuous cycles of engagement, analysis, feedback, and reflection rather than as a linear sequence of activities. Pre-service teachers generate inputs through learning activities, teaching practice, and reflective interaction; the AI system analyzes these inputs to provide personalized feedback and recommendations, while human mentors offer contextual guidance, professional judgment, and socio-emotional support. Importantly, AI does not replace the human mentor but augments the mentoring process, enhancing the quality, consistency, and scalability of support available to pre-service teachers.

### 3.3 Model phases

The model is implemented across three interconnected phases as following:

#### 3.3.1 Pre-practicum phase

This phase focuses on preparing pre-service teachers cognitively and emotionally before entering real classroom environments. Participants engage with instructional content through platforms such as Google Classroom and Nearpod, where they explore teaching strategies, analyze classroom scenarios, and complete guided activities. Reflective discussions are facilitated through platforms such as Facebook and Padlet, allowing participants to express expectations, concerns, and prior beliefs about teaching.

Within this phase, the AI system (e.g., Gemini) functions as a personalized support tool by analyzing participants’ responses and interactions to generate tailored suggestions, recommend relevant learning resources, and provide initial formative feedback. This process supports the development of foundational self-efficacy beliefs and promotes early emotional awareness by addressing concerns and anticipatory anxiety.

#### 3.3.2 During practicum phase

During the practicum, the model supports real-time teaching experiences and ongoing professional development. Pre-service teachers implement mini-lessons in authentic classroom settings and document their experiences through multimedia artifacts (e.g., videos, reflections, and teaching logs). These experiences are shared and discussed through online platforms, enabling peer interaction and collaborative problem-solving.

At this stage, AI plays a critical role in analyzing teaching-related inputs and modifying patterns related to instructional challenges, classroom management, and emotional responses. Based on this analysis, the system provides immediate, context-sensitive feedback and suggests alternative instructional strategies. The integration of platforms such as Nearpod further enables the design of interactive learning activities, while AI-generated insights support adaptive decision-making. This phase is particularly central to strengthening self-efficacy through mastery experiences and enhancing emotional intelligence through real-time reflection and regulation.

#### 3.3.3 Post-practicum phase

The post-practicum phase emphasizes reflection, evaluation, and future development. Pre-service teachers engage in structured reflection by producing final reports, sharing teaching artifacts, and discussing their experiences with peers and mentors. These reflective activities are facilitated through the same digital platforms to ensure continuity and accessibility.

The AI system synthesizes accumulated data from previous phases to generate comprehensive feedback on participants’ progress, including indicators related to self-efficacy and emotional development. It also provides personalized recommendations for further professional growth and identifies areas requiring additional support. These insights are shared with both pre-service teachers and human mentors, enabling more informed and targeted guidance. This phase consolidates learning experiences and supports the transition from guided practice to autonomous professional development.

### 3.4 AI-supported adaptive mentoring mechanisms

Within the model, AI serves as a core mechanism that enhances mentoring through three primary functions: adaptive feedback, personalization, and continuous monitoring. First, AI provides real-time, formative feedback based on participants’ inputs, reducing delays typically associated with traditional mentoring. Second, it enables personalized support by tailoring recommendations to individual needs, performance levels, and learning preferences. Third, AI facilitates longitudinal monitoring by tracking patterns in participants’ engagement, reflections, and performance over time. These capabilities allow the system to detect indicators related to stress, confidence, and instructional challenges, thereby supporting both cognitive and emotional aspects of teacher development.

**Figure 1:**
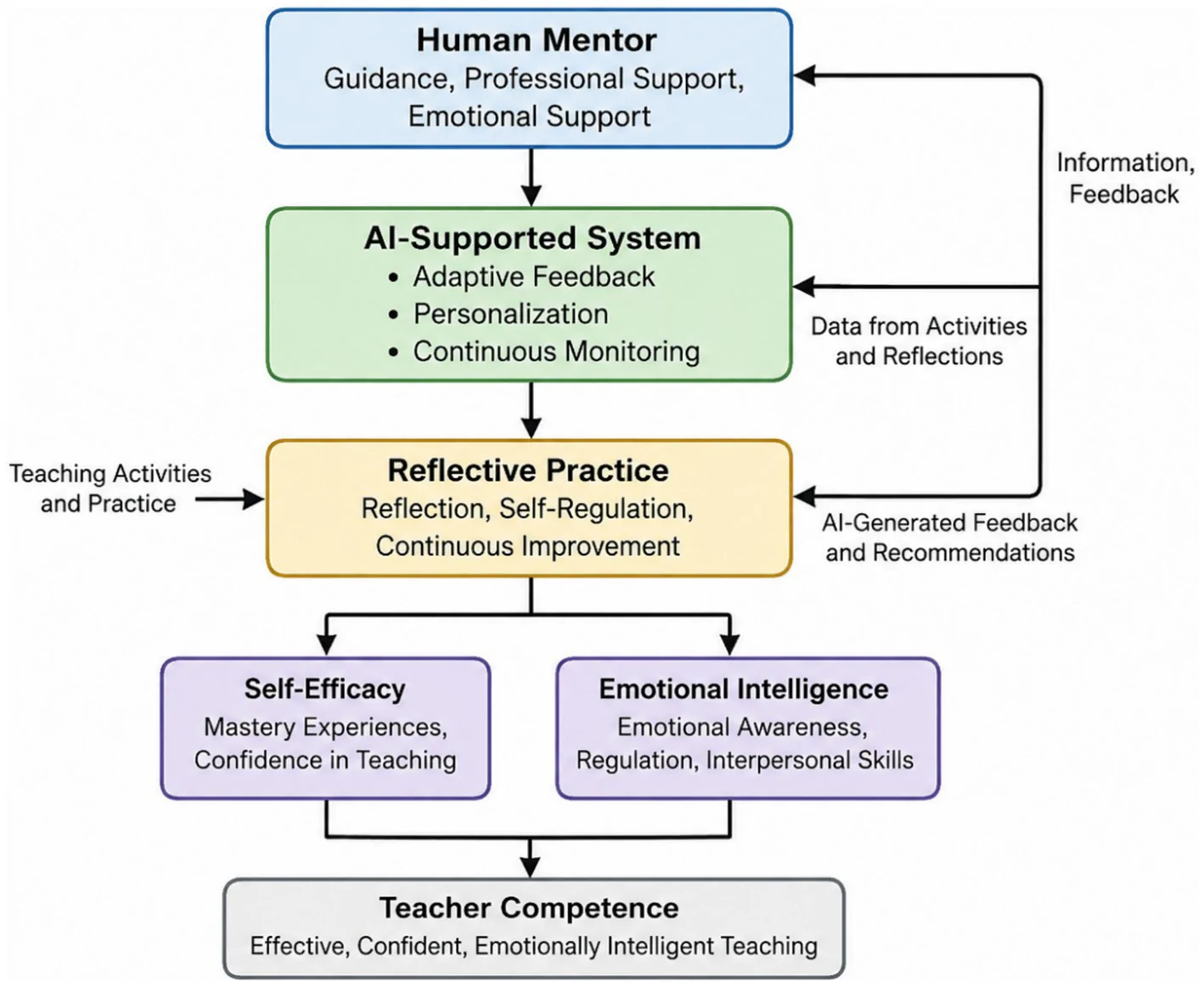
AI-supported e-mentoring model for developing self-efficacy and emotional intelligence among EFL pre-service teachers.

Importantly, AI does not replace the human mentor but rather augments the mentoring process by providing data-informed insights that enhance the quality, consistency, and scalability of support. Thus, the model addresses key limitations of traditional e-mentoring and offers a more responsive and holistic approach to developing self-efficacy and emotional intelligence in EFL pre-service teachers. The following Figure 1 illustrates the AI-supported E-mentoring model for developing self-efficacy and emotional intelligence among EFL pre-service teachers.

As shown in Figure 1, the model’s three phases operate as an integrated, cyclical system in which human mentoring and AI-driven support jointly sustain the development of self-efficacy and emotional intelligence throughout the practicum experience. The specific procedures, platforms, and weekly structure through which this model was implemented are detailed in the Methodology section.

## 4 Methodology

### 4.1 Research design

This study adopted an embedded mixed-methods design within a quasi-experimental framework to examine the effects of an AI-supported e-mentoring model on EFL prospective teachers’ self-efficacy and emotional intelligence. In an embedded design, one data set plays a supportive role within a study grounded primarily in the other data type (Creswell and Plano Clark, 2018).

In the present study, the quantitative strand, pre- and post-intervention comparisons between an experimental and a control group, served as the primary basis for answering the study’s quantitative research questions (RQ2 and RQ3), while the qualitative strand, consisting of semi-structured interviews and weekly reflective inputs, was embedded within this quasi-experimental structure to provide contextual insight into how and why changes in self-efficacy and emotional intelligence occurred over the course of the intervention. This design was considered appropriate given the nature of the study: because the intervention unfolded as a dynamic, iterative mentoring process rather than a fixed instructional sequence, numerical pre-post comparisons alone could establish whether change occurred, but could not capture the mechanisms, classroom experiences, and reflective processes underlying that change.

Embedding qualitative data collection throughout the practicum period therefore allowed the study to document participants’ evolving beliefs and practices as they happened, providing explanatory depth that complements, rather than duplicates, the quantitative findings.

The design incorporated pre- and post-intervention measurements with an experimental group receiving the AI-supported e-mentoring intervention and a control group following standard practicum procedures. Quantitative data were collected through pre- and post-administration of the Teachers’ Sense of Efficacy Scale (TSES) and the Trait Emotional Intelligence Questionnaire (TEIQue). Qualitative data were gathered via semi-structured interviews and weekly reflective inputs on the e-mentoring platforms (Google Classroom, Nearpod, Facebook, Padlet).

The experimental group participated in the AI-supported e-mentoring model, which included pre-practicum, during-practicum, and post-practicum stages. Prospective teachers in the experimental group actively engaged with the AI system (Gemini), which provided personalized suggestions, adaptive feedback, and continuous monitoring. The researcher performed a dual role as a mentor and facilitator, guiding the process and supporting reflective practices, in addition to observing participants’ development throughout the intervention.

The control group, on the other hand, followed the standard practicum procedures implemented in the Faculty of Education. Participants received school-based supervision without access to the AI-supported e-mentoring system or structured digital mentoring platforms. Supervision was provided within the school context primarily by the head of the English department and the cooperating classroom teacher, with occasional academic follow-up from curriculum department supervisors. Feedback was delivered in an informal and predominantly oral manner during or after lesson implementation, focusing mainly on lesson delivery and classroom management issues. Communication between supervisors and student teachers occurred on an as-needed basis rather than through scheduled mentoring cycles, and no structured online platforms or AI tools were used. In some cases, participants also used WhatsApp for communication; however, it was not used as a structured instructional or reflective learning tool. The control condition therefore represented the typical institutional practicum model without systematic e-mentoring or technology-enhanced feedback.

The intervention was directly implemented by the first author, who assumed primary responsibility for mentoring, classroom-based application, and data collection throughout the study, under the guidance of the second and third authors.

### 4.2 Participants

The present study targeted second-year EFL pre-service teachers enrolled in the Faculty of Education, Ain Shams University, during the first semester of the academic year (2025–2026). All participants were placed in public schools as part of their practicum training, representing their first exposure to real classroom teaching. School placement for the practicum was determined by the Faculty of Education prior to the study and was outside the researcher’s control.

A total of 50 pre-service teachers participated in the study and were distributed across 10 public schools, with approximately five pre-service teachers in each school. Randomization was conducted at the school level rather than the individual level: once the 10 practicum schools had been designated by the Faculty, the researcher randomly assigned the schools, as complete units, to the experimental condition (5 schools; *n* = 25 pre-service teachers) or the control condition (5 schools; *n* = 25 pre-service teachers) using a simple lottery method. All pre-service teachers within a given school were therefore assigned to the same condition.

All participants were females due to accessibility and practicum placement constraints. Within the practicum system, pre-service teachers are typically placed in gender-segregated schools, with female pre-service teachers assigned to girls’ schools and male pre-service teachers assigned to boys’ schools. The implementation of the intervention required school visits, classroom observation, mentoring, and follow-up within the practicum sites; therefore, access to male schools was limited by administrative regulations and prevailing cultural norms.

The sample size was determined by the number of pre-service teachers available during the practicum period and was constrained by institutional placement conditions. Because the sample was determined by availability rather than an *a priori* power analysis, a sensitivity analysis was conducted using G*Power 3.1. Assuming a two-tailed independent-samples *t*-test, *α* = 0.05, and power = 0.80, the minimum detectable effect size for *N* = 50 was Cohen’s d = 0.81 (a large effect). The study was therefore adequately powered to detect large intervention effects, whereas small-to-moderate effects may have gone undetected, a limitation considered further in the Limitations section.

The selection of participants was based on several considerations. First, pre-service teachers at this stage possess foundational theoretical knowledge but limited practical teaching experience in authentic classroom contexts. Second, as this was their first practicum experience, they had not previously engaged in real teaching situations involving classroom management, supervision, or instructional delivery challenges. Third, this stage represents a critical transition from theory to practice, during which structured pedagogical and emotional support is essential for developing teaching competence, self-efficacy, and emotional intelligence.

### 4.3 Instruments

#### 4.3.1 Semi-structured interviews

Semi-structured interviews were selected as the primary qualitative instrument because they combine a predetermined set of guiding questions with sufficient flexibility to probe participants’ responses in depth, allowing rich, participant-driven accounts to emerge while still ensuring comparability across interviews (Kvale and Brinkmann, 2009). This flexibility made the technique particularly suitable for capturing participants’ perceptions, beliefs, and experiences before and after the AI-supported e-mentoring intervention.

The pre-intervention interview (Appendix A) aimed to identify participants’ baseline beliefs about classroom management, student engagement, and instructional practices, and to ensure that the components of the e-mentoring model aligned with their needs. Six questions targeted classroom management and student engagement, while five questions focused on instructional practices.

The post-intervention interview (Appendix B) was administered after the intervention to explore changes in participants’ beliefs, ideas, and confidence, and to obtain feedback on the AI-supported e-mentoring model. Nine questions were used, addressing classroom management, student engagement, instructional practices, and the perceived benefits of the training. The semi-structured format allowed for flexibility in probing participants’ responses and capturing rich qualitative data on the development of self-efficacy, emotional intelligence, and instructional competence. The interviews were conducted individually, recorded with participants’ consent, and analyzed thematically to identify patterns of change and the impact of the intervention.

#### 4.3.2 Teacher’s sense of efficacy scale (TSES)

TSES (Appendix C) was employed to assess the impact of the AI-supported e-mentoring model on participants’ self-efficacy. The scale focuses on three core dimensions of teaching efficacy: classroom management, student engagement, and instructional practices. The long form of the scale used in this study consists of 24 items, divided equally across these three dimensions, with responses provided on a 5-point Likert scale (1 = Nothing, 5 = A Great Deal).

Participants completed the scale twice: once prior to the intervention and once after completing the 10-week AI-supported e-mentoring program. Instructions were provided, and participants had approximately 1 hour to respond to all items. This pre- and post-administration allowed the researcher to measure changes in perceived self-efficacy across the three teaching domains.

##### 4.3.2.1 Reliability and validity

The TSES has demonstrated high reliability in multiple validation studies conducted by Tschannen-Moran and Hoy (2001). Internal consistency coefficients reported for the long form were 0.91 for instructional practices, 0.90 for classroom management, 0.87 for student engagement, and 0.94 for the overall 24-item scale. Validity was established through positive correlations with other teacher efficacy measures, including the Gibson and Dembo Teacher Efficacy Scale, providing prior evidence that the scale accurately captures teachers’ perceived effectiveness across the three main teaching domains.

In the current study, internal consistency was also examined using the present sample. Cronbach’s alpha and McDonald’s omega were calculated for the total TSES scale and its three subscales at both pre-test and post-test (see Table 1). Reliability coefficients ranged from 0.84 to 0.90 (*α*) and 0.85 to 0.91 (*ω*) at pre-test, and from 0.88 to 0.96 (*α*) and 0.89 to 0.96 (*ω*) at post-test, indicating good to excellent internal consistency across both administrations.

**Table 1:** Internal consistency of the TSES total scale and subscales at pre-test and post-test.

Scale	Items	Pre-test *α*	Pre-test *ω*	Post-test *α*	Post-test *ω*
Classroom management	8	0.86	0.87	0.89	0.90
Student engagement	8	0.84	0.85	0.88	0.89
Instructional practices	8	0.90	0.91	0.92	0.93
Total TSES	24	0.94	0.95	0.96	0.96

#### 4.3.3 Trait emotional intelligence questionnaire (TEIQue)

The TEIQue (Petrides, 2009) was used to assess pre-service EFL teachers’ emotional intelligence before and after the AI-supported e-mentoring intervention (Appendix D). The questionnaire consists of 30 items covering 15 facets of trait emotional intelligence, grouped under four factors: Emotionality, Sociability, Well-Being, and Self-Control. Each facet is represented by two items, and participants responded using a 7-point Likert scale (1 = Completely Disagree, 7 = Completely Agree). Participants completed the TEIQue twice: once prior to the practicum and intervention, and again at the end of the intervention.

Additionally, both the TSES and the TEIQue were administered in their original English form, with no translation, item modification, or changes to the response format, given that participants were EFL pre-service teachers with advanced English proficiency.

##### 4.3.3.1 Validity and reliability

Previous studies (Azghandi et al., 2007) have confirmed the TEIQue as a reliable and valid instrument for measuring trait emotional intelligence. Internal consistency coefficients were acceptable (*α* = 0.76), and test–retest reliability was satisfactory (*r* = 0.71). Factor analysis supported the structure of the four factors, demonstrating the questionnaire’s ability to capture the main aspects of trait emotional intelligence. This scale was therefore deemed suitable for evaluating the effect of the AI-supported e-mentoring model on participants’ emotional intelligence.

### 4.4 Outline of the AI-supported E-mentoring intervention

The AI-supported e-mentoring intervention was conducted over 10 weeks, totaling approximately 35 h, aligned with the practicum schedule, during which prospective teachers attended public schools once per week. The intervention was designed to directly operationalize the theoretical foundations outlined in Section 3.1. The three-phase structure (pre-practicum, during-practicum, post-practicum) was intended to provide repeated mastery experiences and structured feedback cycles consistent with Social Cognitive Theory, while the combination of collaborative digital platforms and AI-generated suggestions was designed to address feedback at different levels, with AI targeting task- and process-level support and human mentoring targeting the self-regulation and self-levels, as described in the feedback framework adopted in this study (Hattie and Timperley, 2007). Rather than following a fixed instructional sequence, the intervention therefore operated as a dynamic, iterative mentoring process guided by a continuous cycle of preparation, application, reflection, and feedback, aiming to enhance participants’ self-efficacy and emotional intelligence.

Before implementation, an on-site orientation familiarized participants with the model’s objectives, procedures, and their roles. A pre-intervention semi-structured interview assessed participants’ initial needs, beliefs, and concerns; the same interview was repeated post-intervention to track development and collect feedback on the mentoring process.

At the practicum’s outset, no predetermined instructional content was provided. Participants engaged in reflective observation of their classroom experiences, identifying challenges encountered in real teaching situations. Based on these reflections, the researcher designed and adapted mentoring support responsive to emerging needs.

Prior to each practicum day, participants completed preparatory activities via Google Classroom, receiving tailored materials addressing previously identified challenges. These included videos on learning styles, strategies for student engagement and motivation, and links to freely available teaching resources. Interactive content and practical demonstrations were delivered via Nearpod to support lesson planning. Gemini was employed to design lesson plans, generate instructional activities, and adapt teaching to students’ levels within specific classroom contexts.

AI interactions followed a general guiding structure that was consistent across all participants, but not a fixed or scripted prompt template: participants were guided to include three elements in their prompts, a brief description of the classroom context, the specific instructional challenge or lesson content involved, and the type of support requested (e.g., alternative activities, classroom management strategies, or ways to explain a grammar or vocabulary point). Within this shared structure, participants formulated their prompts individually and in their own words, reflecting their own classroom experiences and language, and inputted lesson plans, instructional challenges, and reflective descriptions of classroom experiences. In response, Gemini generated pedagogical suggestions, including alternative lesson structures, classroom activities, games, and classroom management strategies tailored to students’ needs. Participants also used the system to refine lesson plans and explore different ways of explaining grammar and vocabulary in more communicative and student-centred ways. This approach was intended to standardize the type of information exchanged with the AI system across the group, while allowing the content of each interaction to remain authentic and responsive to each participant’s specific teaching context.

AI interactions occurred on a weekly basis throughout the intervention and primarily provided immediate, strategy-oriented suggestions. This differed from human mentoring, which focused on contextualized feedback, classroom observation, and deeper reflective discussion. Accordingly, AI functioned as a rapid instructional support tool, while the researcher provided interpretive feedback and socio-emotional guidance based on actual classroom practice.

The intervention incorporated multiple digital platforms because each served a distinct pedagogical and mentoring function within the e-mentoring process rather than acting as independent intervention components. For instance, Google Classroom functioned as the main organizational platform through which participants accessed weekly tasks, instructional videos, and learning resources. Nearpod was used to deliver interactive presentations, quizzes, and demonstrations of instructional activities and lesson design examples. Facebook served as a collaborative mentoring space where participants shared teaching videos, discussed classroom challenges, and exchanged peer and mentor feedback. Padlet was employed primarily for anonymous reflection and scenario-based analysis, enabling participants to discuss classroom situations and consider alternative responses. Accordingly, the use of multiple platforms was intended to create an integrated mentoring environment and avoid repetitive interaction through a single tool during the practicum period.

During the practicum, participants applied suggested strategies in authentic classrooms and documented their experiences through notes, photos, or short videos. Reflections were shared in a Facebook group for discussion, peer feedback, and collaborative problem-solving. AI (Gemini) provided immediate guidance and context-sensitive suggestions.

Post-practicum reflection continued via Padlet, where participants engaged with common classroom scenarios derived from their shared experiences. They analyzed situations, proposed alternative strategies, and critically evaluated their decisions. AI tools supported deeper reflection by allowing comparison of different approaches and assessment of instructional choices’ strengths and limitations.

Throughout the intervention, the researcher acted as mentor and facilitator, monitoring reflections, guiding discussions, and providing individualized feedback. Regular online meetings via Google Classroom offered a supportive space to discuss pedagogical and emotional challenges, share experiences, and receive guidance on managing practicum-related stress.

The intervention functioned as an ongoing mentoring cycle: participants identified challenges, explored solutions, implemented strategies in classrooms, and reflected on their experiences. Mentoring support evolved based on participants’ needs. Initial reflections revealed challenges with student engagement and emotional awareness, which were addressed through targeted support. Subsequent needs emerged, including classroom management and instructional practices such as teaching vocabulary in context and communicative grammar, which were incorporated as the process progressed.

Participants uploaded recorded teaching videos after implementing strategies, serving as a basis for reflection-in-action. They analyzed their performance, identified strengths and areas for improvement, and evaluated the effectiveness of different strategies. The researcher provided detailed constructive feedback to refine practice. The weekly intervention structure is summarized in Table 2.

**Table 2:** Weekly outline of the AI-supported e-mentoring intervention.

Week	Session title	Main focus
0	Orientation	Introduction to the model and pre-interview
1	Student Engagement and Emotional Awareness	Motivation and classroom emotions
2	Classroom Management	Rules, procedures, and handling challenges
3	Building Teaching Confidence	Practice and feedback
4	Teaching Vocabulary in Context	Meaning, form, and engagement
5	Teaching Grammar Communicatively	Communicative grammar strategies
6	Teaching Reading and Writing	Instructional strategies
7	Teaching Listening and Speaking	Communication and interaction
8	Communicative Activities and Interaction	Games and student participation
9	Reflective Practice and Emotional Regulation	Managing stress and reflection
10	Reflecting on Teaching Experience and Future Improvement	Final reflection and post-interview

### 4.5 Data analysis

A mixed-methods approach was used to analyse quantitative and qualitative data. Quantitative analyses were conducted using SPSS (Version 25). Prior to examining the intervention effects, baseline equivalence between the experimental and control groups was assessed using independent-samples t-tests on pre-intervention scores of the Teachers’ Sense of Efficacy Scale (TSES) and the Trait Emotional Intelligence Questionnaire (TEIQue). This analysis was conducted to ensure comparability between groups before implementing the intervention.

To examine the effect of the AI-supported e-mentoring intervention on self-efficacy and emotional intelligence, a series of 2 (Group: experimental vs. control) × 2 (Time: pre-test vs. post-test) mixed ANOVAs were conducted for each outcome measure (TSES and TEIQue), with Group as the between-subjects factor and Time as the within-subjects factor. This approach was selected because it simultaneously models within-subject change over time and between-group differences, and directly tests whether the two groups changed differently across time via the Time × Group interaction term, the effect of central interest in this pre-post, two-group design. Unlike ANCOVA, which assumes homogeneity of regression slopes across groups, the mixed ANOVA does not require this assumption and provides both within-group (pre-to-post) and between-group effect sizes directly relevant to the study’s research questions. The same procedure was applied to each TSES subdomain (classroom management, student engagement, and instructional practices) to provide a more detailed understanding of the intervention’s effects across specific dimensions of teaching efficacy. Effect sizes were calculated using partial eta-squared (*η*^2^p) for the omnibus tests and Cohen’s d for pairwise (within- and between-group) comparisons, with values interpreted according to conventional benchmarks for small, medium, and large effects (Cohen, 1988).

Qualitative data obtained from the semi-structured interviews were analyzed using thematic analysis following Braun and Clarke (2006), applying a hybrid coding approach. Coding was conducted at the level of meaning units (sentences and phrases), and initial coding categories were informed by the study’s theoretical framework (the TSES domains and TEIQue factors) while remaining open to codes emerging inductively from participants’ own accounts. Coding was conducted collaboratively with another Applied Linguistics professor to enhance reliability and trustworthiness. All researchers independently double-coded all 50 interview transcripts, and inter-rater reliability was assessed using Cohen’s kappa, yielding a value of *κ* = 0.84, indicating almost strong agreement. Any coding discrepancies were subsequently discussed and reconciled through consensus, thereby strengthening the credibility and trustworthiness of the qualitative findings.

Consistent with the embedded mixed-methods design, the quantitative and qualitative strands were integrated following established principles for mixed-methods integration, in which qualitative findings are used to explain, contextualize, and elaborate upon quantitative results rather than being analyzed as an entirely separate data set (Fetters et al., 2013). In practice, this meant that thematic patterns identified in the interviews and reflective logs were systematically compared against the statistically significant quantitative gains in self-efficacy and emotional intelligence, in order to explain the mechanisms underlying these changes.

## 5 Results

### 5.1 Quantitative results

#### 5.1.1 Baseline equivalence between groups

Prior to implementing the AI-supported e-mentoring intervention, independent-samples t-tests were conducted to determine whether the experimental and control groups differed significantly in their pre-intervention levels of self-efficacy and emotional intelligence. Establishing baseline equivalence was important to ensure that the two groups were comparable before the intervention and that subsequent differences could be interpreted in relation to the intervention rather than substantial pre-existing group differences. As shown in Table 3, the experimental and control groups had very similar mean scores on both measures at pre-intervention.

**Table 3:** Baseline equivalence of the experimental and control groups on pre-intervention measures.

Variable	Experimental (*n* = 25), M ± SD	Control (*n* = 25), M ± SD	*t*	*df*	*p*
Self-efficacy (TSES)	89.76 ± 10.12	88.92 ± 9.84	0.30	48	0.76
Emotional intelligence (TEIQue)	121.84 ± 11.36	120.96 ± 10.91	0.28	48	0.78

The results presented in Table 3 indicate that there was no statistically significant difference between the experimental and control groups in pre-intervention self-efficacy, t(48) = 0.30, *p* = .76. Similarly, the groups did not differ significantly in their pre-intervention emotional intelligence scores, t(48) = 0.28, *p* = .78. Thus, the two groups were statistically comparable at baseline on both outcome measures, providing an appropriate basis for examining changes following the intervention.

#### 5.1.2 Self-efficacy: effects of group and time

To answer RQ2, a 2 (Group: experimental, control) × 2 (Time: pre-intervention, post-intervention) mixed ANOVA was conducted to determine whether self-efficacy scores changed differently over time across the two groups. The analysis focused primarily on the Time × Group interaction, which indicates whether the amount of change from pre- to post-intervention differed significantly between the experimental and control groups.

The descriptive statistics for self-efficacy are presented in Table 4. The experimental group increased from a pre-intervention mean of 89.76 (SD = 10.12) to a post-intervention mean of 112.40 (SD = 8.23). In comparison, the control group increased from 88.92 (SD = 9.84) to 102.16 (SD = 9.12).

**Table 4:** Descriptive statistics for self-efficacy (TSES) scores by group and time.

Group	Pre-Intervention M ± SD	Post-Intervention M ± SD
Experimental	89.76 ± 10.12	112.40 ± 8.23
Control	88.92 ± 9.84	102.16 ± 9.12

As shown in Table 4, both groups demonstrated higher self-efficacy scores at post-intervention; however, the increase was substantially greater in the experimental group. To determine whether these changes were statistically significant and whether the pattern of change differed between groups, the mixed ANOVA results are presented in Table 5.

**Table 5:** Mixed ANOVA results for self-efficacy (TSES) scores.

Source	*df*	*F*	*p*	Partial *η*^2^
Time	1, 48	88.4	<0.001	0.65
Group	1, 48	15.2	<0.001	0.24
Time × Group	1, 48	32.7	<0.001	0.41

As indicated in Table 5, the main effect of Time was statistically significant, *F*(1, 48) = 88.4, *p* < .001, partial *η*² = .65, indicating an overall increase in self-efficacy scores across the two measurement occasions. The main effect of Group was also statistically significant, *F*(1, 48) = 15.2, *p* < .001, partial *η*² = .24. Most importantly, the Time × Group interaction was statistically significant, *F*(1, 48) = 32.7, *p* < .001, partial *η*² = .41.

The significant Time × Group interaction demonstrates that the change in self-efficacy over time differed significantly between the experimental and control groups. In particular, the experimental group demonstrated a substantially greater improvement than the control group, providing an affirmative answer to RQ2. Follow-up simple-effects analysis further confirmed a significant pre- to post-intervention increase in the experimental group, whereas the increase observed in the control group was small and not statistically significant.

The magnitude of the observed improvement also indicates a substantial practical gain in perceived teaching efficacy. Given the TSES total score range of 24–120, the experimental group moved from a moderate level of perceived efficacy toward a high-efficacy profile following the intervention. This improvement was reflected in greater confidence in areas such as managing disruptive behaviour, promoting student engagement, and independently applying instructional strategies.

#### 5.1.3 Self-efficacy subdomains: effects of group and time

To examine whether the intervention influenced specific dimensions of teaching self-efficacy, separate 2 (Group: experimental, control) × 2 (Time: pre-intervention, post-intervention) mixed ANOVAs were conducted for the three TSES subdomains: classroom management, student engagement, and instructional practices.

The descriptive statistics for the three subdomains are presented in Table 6. As shown in the table, the experimental group demonstrated increases across all three domains from pre- to post-intervention. Classroom management scores increased from 29.80 (SD = 4.10) to 37.90 (SD = 3.85), student engagement scores increased from 28.45 (SD = 3.95) to 36.80 (SD = 3.70), and instructional practices scores increased from 31.51 (SD = 4.25) to 37.70 (SD = 3.90). The control group also showed increases, although these were considerably smaller.

**Table 6:** Descriptive statistics for TSES subdomains by group and time.

Subdomain	Group	Pre-Intervention M ± SD	Post-Intervention M ± SD
Classroom management	Experimental	29.80 ± 4.10	37.90 ± 3.85
Classroom management	Control	29.50 ± 4.00	31.20 ± 3.90
Student engagement	Experimental	28.45 ± 3.95	36.80 ± 3.70
Student engagement	Control	28.10 ± 3.80	30.50 ± 3.75
Instructional practices	Experimental	31.51 ± 4.25	37.70 ± 3.90
Instructional practices	Control	31.30 ± 4.10	33.10 ± 4.00

The descriptive pattern in Table 6 suggests that the experimental group experienced greater gains across all three self-efficacy subdomains. To test whether these differences in change were statistically significant, the Time × Group interaction effects from the three mixed ANOVAs are reported in Table 7.

**Table 7:** Mixed ANOVA results for TSES subdomains: time × group interactions.

Subdomain	*df*	*F*	*p*	Partial *η*^2^
Classroom Management	1, 48	24.1	<0.001	0.33
Student Engagement	1, 48	27.8	<0.001	0.37
Instructional Practices	1, 48	19.5	<0.001	0.29

As shown in Table 7, the Time × Group interaction was statistically significant for classroom management, *F*(1, 48) = 24.1, *p* < .001, partial *η*² = .33; student engagement, *F*(1, 48) = 27.8, *p* < .001, partial *η*² = .37; and instructional practices, *F*(1, 48) = 19.5, *p* < .001, partial *η*² = .29.

These findings indicate that the experimental group improved significantly more than the control group across all three dimensions of teaching self-efficacy. Based on the partial *η*² values, the largest interaction effect was observed for student engagement (partial *η*² = .37), followed by classroom management (partial *η*² = .33) and instructional practices (partial *η*² = .29). Thus, the intervention appears to have strengthened not only participants’ overall sense of teaching efficacy but also specific dimensions of their perceived pedagogical competence.

The quantitative pattern is also consistent with the qualitative findings, in which participants described developing greater confidence in classroom management, adopting more student-centred engagement techniques, and becoming more capable of applying instructional strategies through the communicative and reflective activities embedded in the intervention.

#### 5.1.4 Emotional intelligence: effects of group and time

To answer RQ3, a 2 (Group: experimental, control) × 2 (Time: pre-intervention, post-intervention) mixed ANOVA was conducted to examine whether emotional intelligence (TEIQue) scores changed significantly over time and whether the magnitude of change differed between the experimental and control groups.

The descriptive statistics for emotional intelligence are presented in Table 8. The experimental group increased from a pre-intervention mean of 121.84 (SD = 11.36) to a post-intervention mean of 135.60 (SD = 10.45). The control group, in comparison, increased from 120.96 (SD = 10.91) to 124.32 (SD = 11.08).

**Table 8:** Descriptive statistics for emotional intelligence (TEIQue) scores by group and time.

Group	Pre M ± SD	Post M ± SD
Experimental	121.84 ± 11.36	135.60 ± 10.45
Control	120.96 ± 10.91	124.32 ± 11.08

As shown in Table 8, both groups demonstrated higher emotional intelligence scores at post-intervention, but the increase was substantially greater in the experimental group. The mixed ANOVA results used to determine whether this pattern of change was statistically significant are presented in Table 9.

**Table 9:** Mixed ANOVA results for emotional intelligence (TEIQue) scores.

Source	*df*	*F*	*p*	Partial *η*^2^
Time	1, 48	61.3	<0.001	0.56
Group	1, 48	11.7	0.001	0.20
Time × group	1, 48	22.4	<0.001	0.32

The results in Table 9 show a statistically significant main effect of Time, *F*(1, 48) = 61.3, *p* < .001, partial *η*² = .56, indicating an overall increase in emotional intelligence across the two measurement occasions. The main effect of Group was also statistically significant, *F*(1, 48) = 11.7, *p* = .001, partial *η*² = .20. Most importantly, the Time × Group interaction was statistically significant, *F*(1, 48) = 22.4, *p* < .001, partial *η*² = .32.

The significant Time × Group interaction indicates that the experimental group experienced a significantly greater improvement in emotional intelligence than the control group, providing an affirmative answer to RQ3. Follow-up simple-effects analysis confirmed a significant increase in emotional intelligence scores in the experimental group from pre- to post-intervention, whereas the increase in the control group was considerably smaller.

The observed improvement may be related to the continuous cycle of reflection, AI-supported feedback, and collaborative peer discussion incorporated into the intervention. These components provided repeated opportunities for participants to reflect on their emotional responses, consider alternative ways of managing teaching-related challenges, and develop more effective interpersonal communication strategies. This interpretation is also consistent with the qualitative findings, which highlighted increased confidence, emotional regulation, and improved coping with classroom challenges among participants.

### 5.2 Qualitative results

#### 5.2.1 Participants’ responses to the semi-structured interview questions

The qualitative data obtained from the semi-structured interviews were analyzed thematically to explore changes in participants’ self-efficacy beliefs, instructional practices, and emotional development following the AI-supported e-mentoring intervention. Four main themes emerged: (1) development of classroom management beliefs, (2) shift toward student-centered engagement, (3) growth in instructional practices and communicative teaching, and (4) development of self-efficacy and emotional intelligence.

##### 5.2.1.1 Theme 1: development of classroom management beliefs

This theme captures how participants’ understanding of classroom management shifted over the course of the intervention, from a control-oriented view toward a more relational, student-centered one. Prior to the intervention, participants demonstrated limited and sometimes inaccurate understanding of classroom management, often associating it with control, silence, and teacher dominance:

*“Classroom management is controlling the students.”*

*“Classroom management is making students silent so that I can explain the lesson without any noise and side talk.”*

These responses reflect a teacher-centered perception of classroom management. After the intervention, participants’ understanding shifted toward more flexible and student-centered perspectives:

*“It means creating activities and competitions to keep students attentive.”*

*“Building good rapport so students can respect me instead of shouting and punishing them.”*

**Figure 2:**
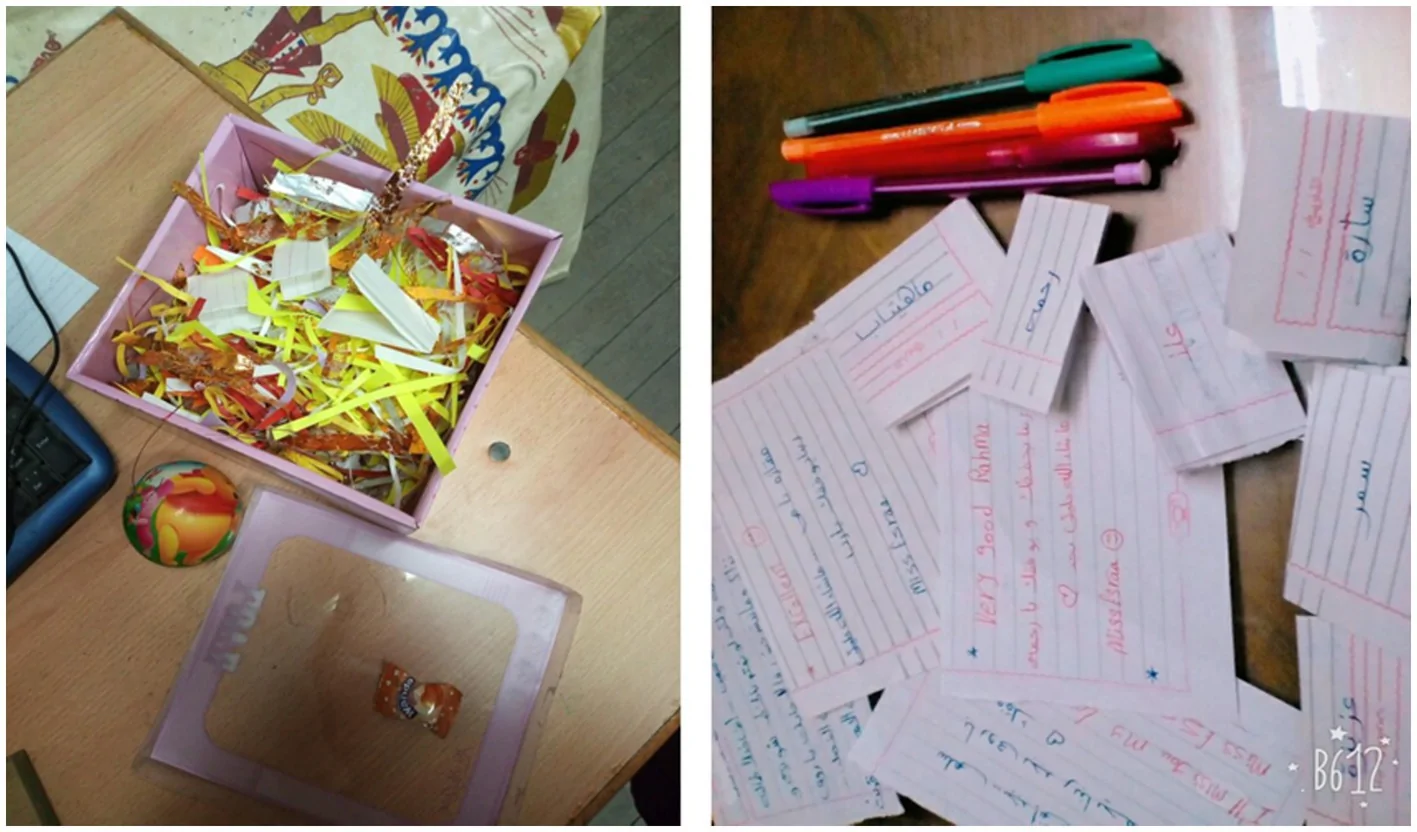
Examples of a flash card with encouraging quotes.

This shift reflects increased awareness of rapport-building as a tool for classroom organization, rather than reliance on control and silence. This development can be attributed to continuous exposure to practical examples, peer discussions, and AI-supported suggestions, which provided participants with useful strategies to handle real classroom situations, build positive relationships, and rethink previous beliefs. For instance, as shown in Figure 2, one of the pre-service teachers created flashcards with encouraging quotes to give students when they achieved something or performed well in class.

##### 5.2.1.2 Theme 2: shift toward student-centered engagement

This theme reflects a change in how participants approached student behavior and engagement, moving from punitive responses toward strategies that actively involve students. Before the intervention, participants relied mainly on punitive and reactive strategies:

*“I will warn the students, then punish them.”*

After the intervention, participants adopted more supportive and engagement-based strategies:

*“I will give students responsibilities keeps them engaged.”*

*“Rewarding them for good behavior and academic achievement to keep them motivated.”*

Participants also demonstrated the ability to adapt strategies to different student personalities. Two participant shared:

*“One of the students was showy and when I made him leader, I won him… instead of shouting at talkative students, I asked them to present role plays and it worked”.*

*“I had one sleepy student in my class. I gave her a task for monitoring her peers and used movement activities to help the sleepy student focus again”.*

**Figure 3:**
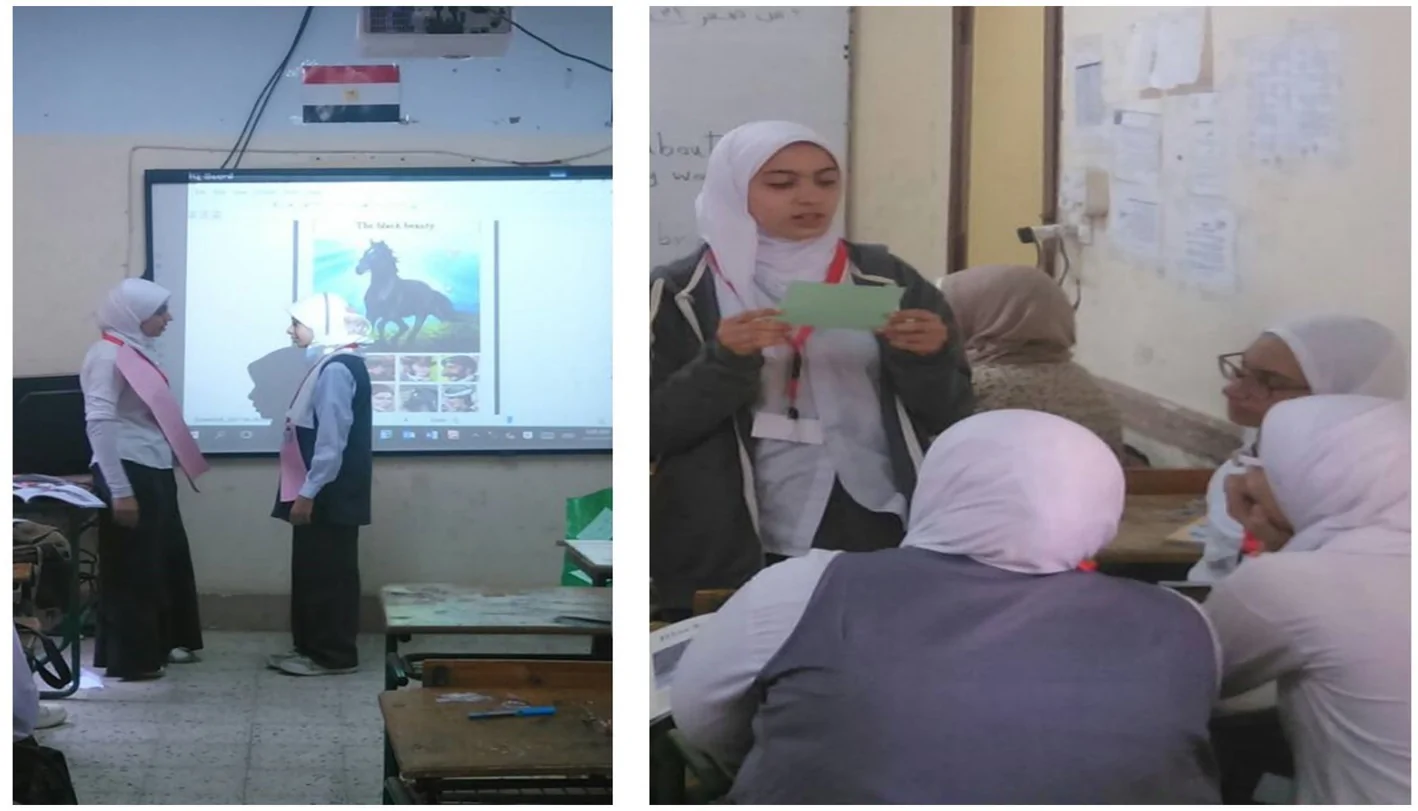
Role-play and group-work activities.

This shift toward engagement and differentiation was further supported by the integration of AI tools and online platforms, which enabled participants to explore and implement alternative instructional strategies, such as interactive activities, competitions, and total physical response tasks (e.g., role-based activities) in their classrooms. Figure 3 shows examples of role-play and group-work activities conducted by students in class.

##### 5.2.1.3 Theme 3: growth in instructional practices and communicative teaching

This theme captures participants’ development from teacher-centered, translation-based instruction toward interactive, communicative teaching methods. Before the intervention, participants’ teaching approaches were limited and teacher-centered:

*“I will explain grammar using rule first and then give examples”.*

*“For difficult vocabulary, I will use Arabic to explain the meaning first, then they will memorize it in English”.*

After the intervention, participants reported using a wider range of interactive and communicative activities:

*“I gave each student a flashcard with a new word. Everyone had to use the words to form correct sentences, which helped them practice today’s lesson without feeling stressed”.*

*“I used a PowerPoint slide showing a house with different rooms (kitchen, bedroom) and objects (sofa, fridge). Students played a game where they had to place the objects in the correct positions using prepositions, for example: (Put the fridge in the kitchen)”.*

**Figure 4:**
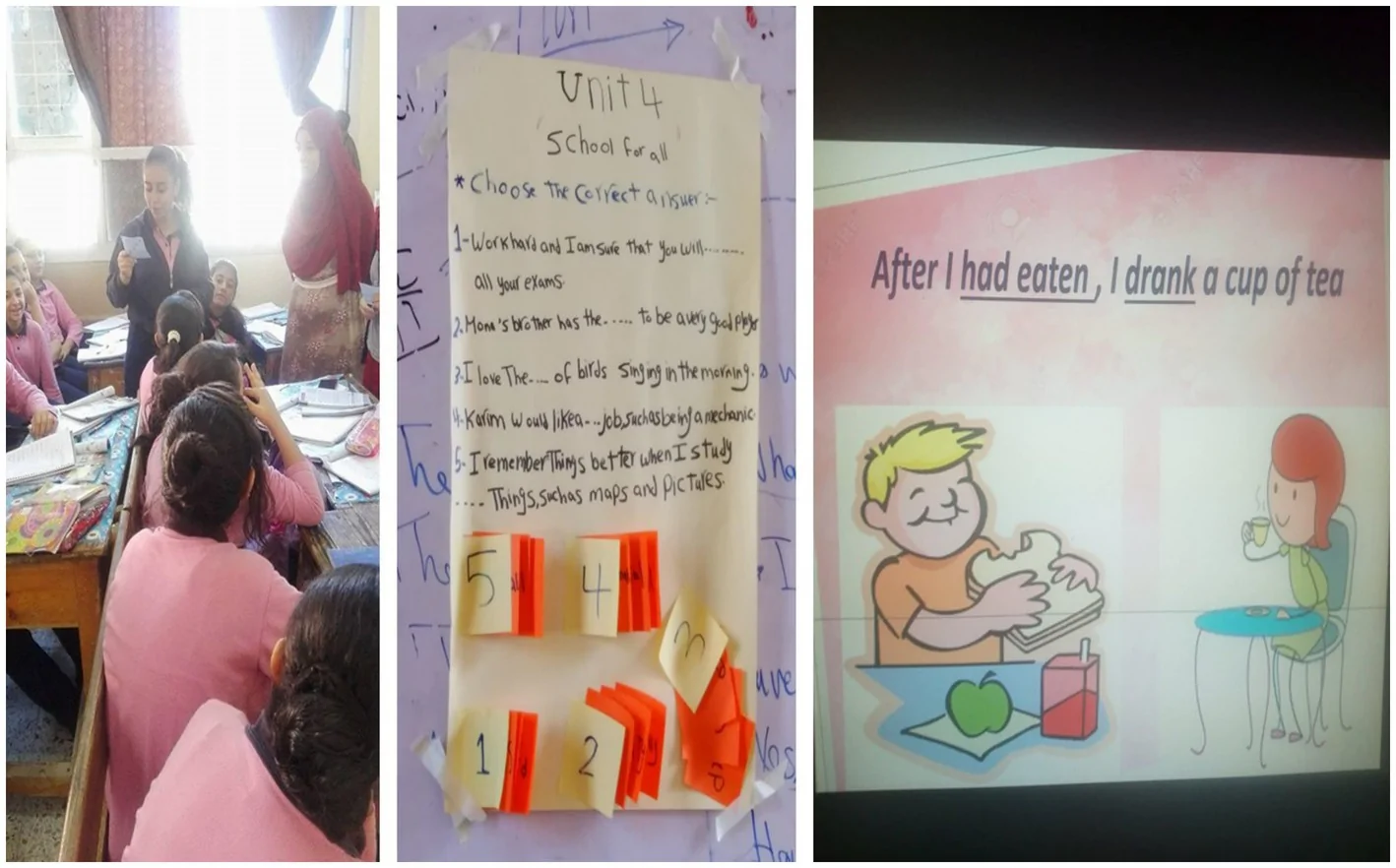
Vocabulary activities conducted by a pre-service teacher using cards and PowerPoint.

The use of AI tools played a supportive role in this process, as participants reported generating lesson ideas, modifying activities to suit students’ levels, and exploring different ways to present content. Figure 4 shows a set of vocabulary activities that one of the pre-service teachers conducted in class using cards and PowerPoint.

##### 5.2.1.4 Theme 4: development of self-efficacy and emotional intelligence

This theme captures participants’ growth in confidence, emotional awareness, and professional identity across the practicum period. Before the intervention, participants expressed fear and lack of confidence:

*“I did not want to be a teacher as I feel afraid of the challenges I will face”.*

After the intervention, participants reported increased confidence and reduced anxiety:

*“When I see my videos while teaching and students’ messages and feedback, I feel happy that I do something and help them in learning”.*

*“I am much more confident about using my body language and even my voice became higher. I do not feel afraid anymore before entering the class”.*

**Figure 5:**
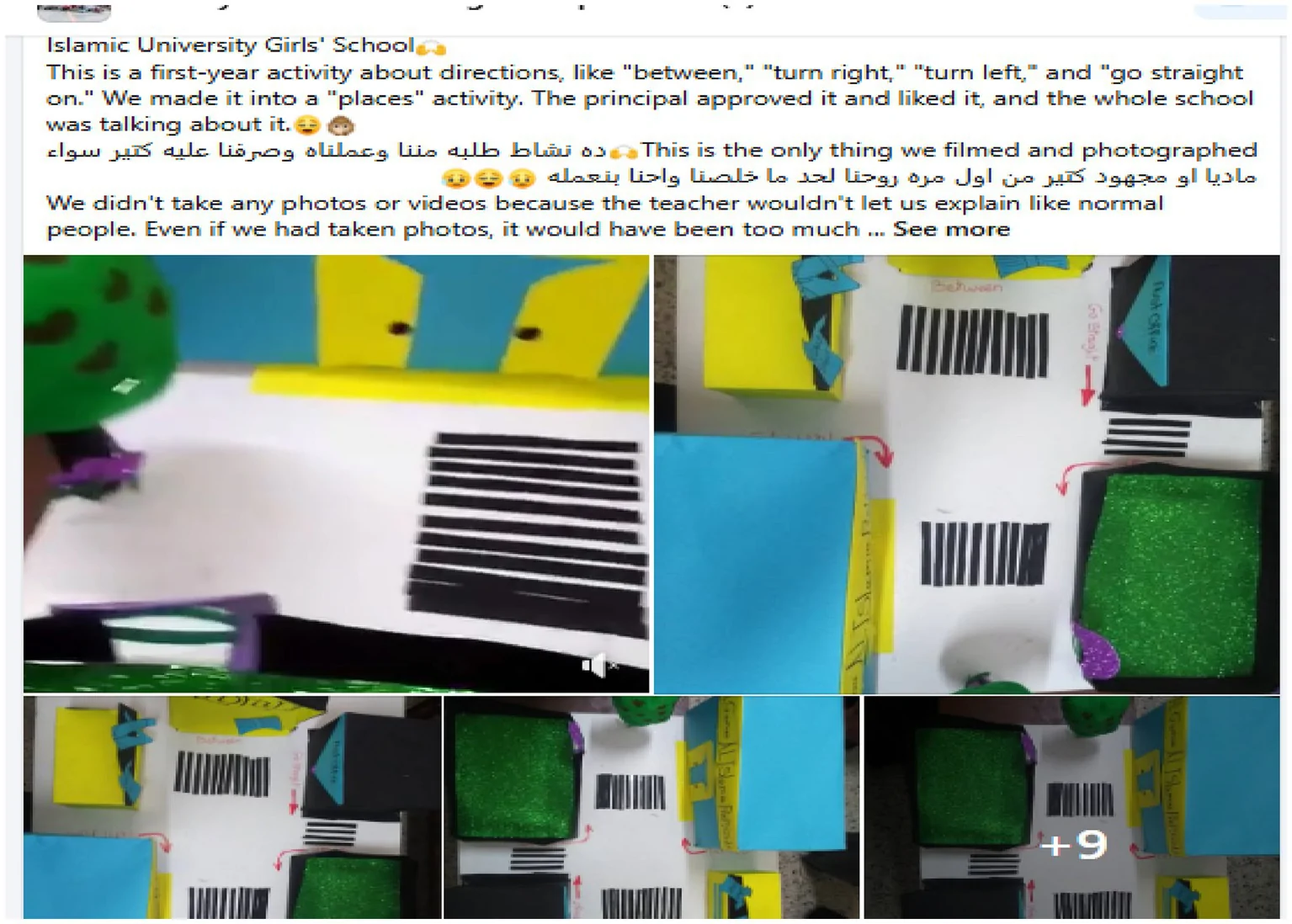
Screenshot of students’ activities and posts shared on Facebook.

This development can be linked to the continuous cycle of reflection, feedback, and practice embedded in the model. Participants used Facebook to share their teaching videos, reflections, and daily activities, gaining feedback from peers and mentors and inspiring others with successful teaching strategies. Figure 5 shows a participant’s post on Facebook, highlighting classroom activities that supported learning and engagement.

### 5.3 Participants’ reflective log

Every week, participants posted the challenges they faced in their practicum on Facebook, along with feedback on applying ideas from AI tools or resources shared via Nearpod and Google Classroom. Weekly online meetings via Google Meet allowed discussion of experiences, effective strategies, and encountered challenges. These activities provided rich insights into how pre-service teachers experienced classroom challenges, collaborated in problem-solving, and developed instructional practices.

#### 5.3.1 Theme 1: reflections on classroom management and student engagement

This theme traces participants’ week-by-week shift from reactive, control-based classroom management toward more reflective, individualized strategies, as the practicum progressed. At the beginning of the practicum, participants’ reflections revealed a strong sense of frustration, confusion, and lack of control when facing real classroom situations for the first time. Their initial posts, shared through the Facebook group, reflected reliance on traditional disciplinary strategies and limited pedagogical awareness:

*“Yesterday was really a tough day! Students do not pay attention even when I raise my voice… they stop talking for a while and then they make noise again. I was depressed and I really hate that class”.*

*“I ordered this naughty student to go to the corner… After few minutes, he came and said sorry to me”.*

These responses indicate that participants initially approached classroom management from a control-oriented perspective, often relying on punishment and authority-based strategies. The first step of building a community of support was done by the researcher. The researcher shared some ideas and sources on how to maintain class management. As interaction increased, participants began to respond to each other’s posts, offering suggestions and sharing similar experiences. While some responses continued to reflect traditional beliefs, others introduced alternative strategies, such as the use of rewards or engagement techniques:

*“Use Games in the class so that they will not find time to talk. Shouting will not work”.*

*“You should reward them using anything like chocolates… they will be happy and participate more”.*

This growing peer interaction helped reduce participants’ sense of isolation and encouraged the exchange of practical classroom strategies. Building on this interaction, more structured guidance was provided through Google Classroom and mentor feedback, which supported a deeper shift in participants’ thinking. For example, when one participant expressed difficulty dealing with a less engaged student, she asked:

*“I faced a problem today. During my lesson, two students kept talking to each other and did not focus on the tasks. I tried asking them to stop several times, but it did not work. What shall I do?”*

The mentor responded by suggesting a more individualized and student-centered approach:

*“Thanks for sharing this situation. In situations like this, try speaking to them privately to understand why they are talking. Then, give them a responsibility related to the lesson, like helping explain a concept to the class or working as group leaders. This will invest their energy positively and make them feel involved rather than punished”.*

Later, the same participant pointed out that one-to-one discussion with students did work with her and helped in building good rapport with students; she explained:

*“I assigned each one to work in a different group and when one of them finished early, I asked her to go and help another group. They focused completely and the lesson went smoothly”.*

This progression, from seeking quick fixes to engaging in reflective, adaptive problem-solving, illustrates how mentor feedback and peer exchange combined to reshape participants’ classroom management approach over the course of the practicum. Some participants also reported using AI tools such as Gemini to explore alternative strategies for similar situations, further expanding their range of responses.

#### 5.3.2 Theme 2: reflections on instructional practices

This theme captures participants’ growing confidence and skill in teaching grammar and vocabulary communicatively, moving away from translation-based methods. At the beginning of the intervention, participants reported limited strategies and reliance on Arabic and traditional explanation methods:

*“I have a problem with explaining the grammar. I mean, how to explain difficult grammar points like present perfect and past simple?”*

These responses highlight a lack of exposure to interactive and student-centered instructional approaches. To address these challenges, participants were provided with resources through Google Classroom, including instructional videos, practical examples, and links to teaching strategies. Nearpod was also used to present interactive content and model how lessons could be designed in more engaging ways. As participants engaged with these materials, their reflections began to demonstrate experimentation with new techniques in their classrooms. For example, one participant described using a game-based activity to increase engagement:

*“I prepared cards with questions and used a paper ball… the student who gets the ball answers the question… the whole class got excited and wanted to participate”.*

Another participant described adopting an inductive approach to teaching grammar:

*“I gave them a story and let them think about the structure… they kept creating sentences until they understood the rule”.*

**Figure 6:**
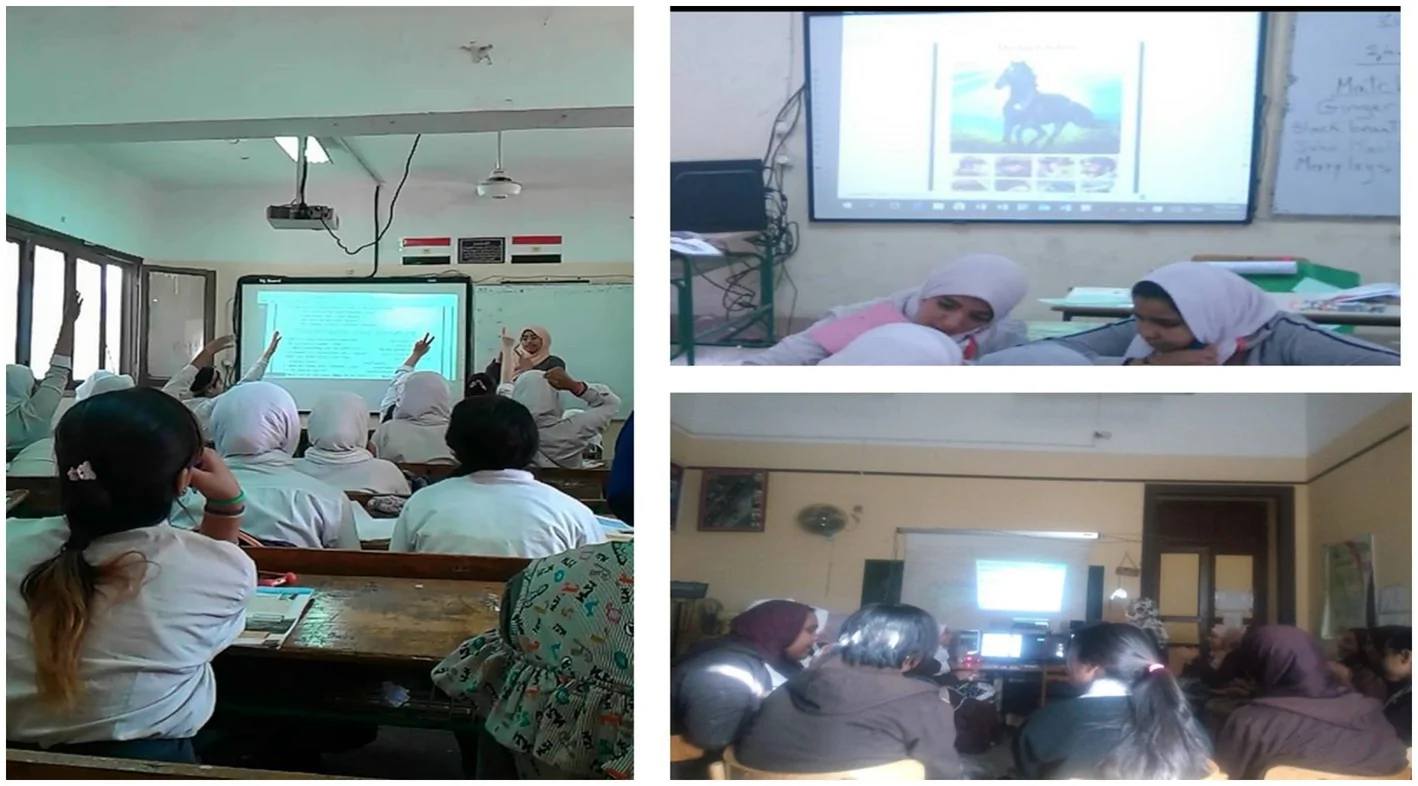
Examples of interactive activities created by pre-service teachers.

These entries show participants not only applying new strategies but actively evaluating their effectiveness based on student response. Figure 6 shows a screenshot of activities and explanations created by the pre-service teachers, illustrating how they used games, group work, and other interactive techniques in class.

Moreover, AI tools such as Gemini supported participants in generating lesson ideas, adapting activities, and finding alternative ways to explain content. These tools were particularly useful in helping participants design communicative activities and adjust their teaching to different student levels and avoid using mother tongue. This was evident in their own descriptions of their teaching:

*“Students were engaged in the animation video that I used from the beginning till the end of the session”.*

*“Students liked the flashcards and their role plays were interesting and funny”.*

Taken together, the reflective logs show that the combination of Google Classroom, Nearpod, Padlet, Facebook, and AI tools created a supportive environment in which participants could test, reflect on, and refine new instructional strategies in response to real classroom situations, contributing to more effective and student-centered teaching over time.

### 5.4 Integration of quantitative and qualitative findings

To understand how the AI-supported e-mentoring intervention produced the quantitative improvements reported above, qualitative findings were integrated with the statistical results. This integration clarifies the mechanisms underlying the development of self-efficacy and emotional intelligence.

The significant Time × Group interaction found for self-efficacy (Table 5) can be explained by participants’ gradual shift from teacher-centered to student-centered pedagogical beliefs, identified across both the interview themes (Section 5.2) and the reflective logs (Section 5.3). Participants initially viewed classroom management as control and discipline, but through continuous engagement with AI-generated suggestions, peer discussion, and mentor feedback, they developed more adaptive strategies such as role assignment, collaborative learning, and engagement-based activities. This progression reflects increased mastery experiences and instructional experimentation, which is reflected quantitatively in the significant gains found across all three TSES subdomains (Table 7).

The significant Time × Group interaction found for emotional intelligence (Table 9) is similarly explained by qualitative evidence of a shift from frustration and emotional instability at the start of the practicum to greater emotional awareness and regulation over time. Weekly reflections, peer discussion, and AI-supported suggestions enabled participants to reinterpret classroom challenges more constructively and develop coping strategies for stressful teaching situations. Collaborative interactions on Facebook and reflective scenario analysis on Padlet, in particular, appear to have contributed to emotional awareness, empathy, and stress regulation.

Overall, the qualitative findings help explain the quantitative improvements by showing that the AI-supported e-mentoring model operated through three interconnected mechanisms: (1) continuous reflective practice, (2) collaborative peer learning, and (3) AI-supported instructional and emotional feedback. Together, these mechanisms account for the measurable gains observed in both self-efficacy and emotional intelligence.

## 6 Discussion

The present study investigated the effect of an AI-supported e-mentoring model on developing EFL pre-service teachers’ self-efficacy and emotional intelligence within the Egyptian practicum context, guided by three research questions: (1) How do EFL pre-service teachers experience and describe changes in their self-efficacy and emotional intelligence throughout the AI-supported e-mentoring model? (2) To what extent does the model improve EFL pre-service teachers’ self-efficacy? (3) To what extent does the model improve EFL pre-service teachers’ emotional intelligence? The findings revealed statistically significant improvements in both variables, with large effect sizes across all comparisons, indicating a strong and practically meaningful impact of the intervention. The following sections address each research question in turn, beginning with self-efficacy and emotional intelligence separately before turning to their combined effect.

### 6.1 Development of self-efficacy

Regarding the second research question, the results demonstrated significant improvement in self-efficacy both between groups (*d* = 1.04) and within the experimental group (*d* = 1.84), indicating the effectiveness of the AI-supported e-mentoring model in enhancing participants’ beliefs in their teaching capabilities. This improvement can be interpreted through the integrated design of the model, which operationalized key mechanisms of Social Cognitive Theory.

Participants actively engaged in authentic classroom teaching, gaining continuous mastery experiences reinforced through structured reflection and iterative application of strategies. Concurrently, vicarious learning and social persuasion occurred via collaborative interactions on digital platforms such as Facebook, Padlet, and Google Classroom. These platforms enabled participants to share challenges, observe peers’ practices, and receive targeted feedback, fostering a sustained and dynamic cycle of professional growth. These findings are consistent with research emphasizing the role of collaborative and digitally mediated professional learning environments in strengthening teacher self-efficacy (Li, 2023; Pan, 2023; Trust and Whalen, 2020).

AI tools, particularly Google Gemini, further extended this process by providing immediate, adaptive, and context-sensitive feedback. This allowed participants to experiment with multiple instructional and classroom management strategies and to refine them iteratively in response to classroom demands. Unlike traditional mentoring, where feedback is often delayed and episodic, AI-supported feedback contributed to more continuous and responsive guidance aligned with participants’ immediate instructional needs. This aligns with recent research highlighting the potential of AI to enhance learners’ engagement, self-efficacy, and instructional confidence in EFL contexts (Samuels et al., 2025; Zhang et al., 2026), as well as the role of perceived support within AI-assisted learning environments in shaping these outcomes (Wang et al., 2025).

Additionally, participants shifted from perceiving teaching primarily as content delivery and classroom control to understanding it as engagement, interaction, and adaptive practice. The integration of tools such as Nearpod supported this transition by modeling interactive pedagogies and narrowing the theory-practice gap. These findings are consistent with prior research indicating that higher self-efficacy is associated with greater adoption of student-centered and innovative instructional approaches (Zhang et al., 2024).

The magnitude of improvement in self-efficacy scores reflects not only statistical significance but also meaningful pedagogical development. Considering the bounded range of the TSES, this increase suggests a shift from partial reliance on external guidance toward a more autonomous and confident teaching profile. This was evident in participants’ classroom practices, where they transitioned from teacher-centered instruction and reliance on disciplinary control to more student-centered approaches, including group work, role-based activities, and interactive task design. Participants also demonstrated greater flexibility in responding to classroom disruptions by using engagement-based strategies rather than punitive approaches. These behavioral changes indicate that gains in self-efficacy were operationalized as observable improvements in classroom teaching competence within authentic instructional settings.

However, these findings should be interpreted with appropriate caution regarding causal attribution. Although AI-supported feedback constituted an important component of the intervention, the observed improvements cannot be attributed solely to AI tools. The intervention was intentionally multi-component, integrating structured mentoring sessions, peer collaboration, reflective practice, and continuous feedback cycles, all of which likely contributed to the observed gains in self-efficacy. Therefore, the findings should be understood as reflecting the collective effect of the AI-supported e-mentoring model rather than the isolated effect of AI. In addition, the control group participated in conventional practicum supervision that was primarily face-to-face, less structured, and did not include systematic online mentoring, AI-supported feedback, or structured reflective cycles; this difference in support structure should be considered when interpreting between-group differences.

### 6.2 Development of emotional intelligence

Regarding the third research question, the findings also revealed significant improvement in emotional intelligence, both between groups (*d* = 0.98) and within the experimental group (*d* = 1.50), indicating that the intervention effectively supported participants’ emotional development. This improvement can be understood in relation to the reflective, collaborative, and supportive structure of the overall mentoring model.

Structured reflective practices, including weekly discussions and posts, provided participants with opportunities to express, analyze, and regulate their emotions in relation to real classroom experiences. Over time, participants demonstrated a shift from frustration, stress, and self-doubt toward more adaptive emotional responses and increased self-awareness. This progression reflects the development of emotional regulation through guided reflection, consistent with established emotional intelligence frameworks (Mayer et al., 2008; Goleman, 1998).

Collaborative engagement further contributed to emotional development by fostering empathy, shared understanding, and interpersonal support. Through interaction on platforms such as Facebook and Padlet, participants engaged with peers’ experiences, exchanged coping strategies, and developed a sense of shared professional identity. These findings align with research highlighting the role of collaborative and reflective environments in enhancing teachers’ emotional competence and resilience (Markowitz and Bouffard, 2025; Talvio and Vuorinen, 2026).

AI-supported input also played a supportive role in this process by offering alternative perspectives on emotionally challenging classroom situations. AI-generated suggestions encouraged more structured, reflective, and student-centered responses, which helped participants reconsider emotionally charged interactions and develop more regulated responses. In this way, AI functioned as one of several mediating tools within a broader socio-collaborative learning environment, rather than as the sole driver of emotional development (Nygren et al., 2025), a pattern broadly consistent with reports that AI-assisted language learning environments can shape learners’ emotional experience, including anxiety, alongside cognitive outcomes (Wang et al., 2024).

Importantly, although large effect sizes were observed for emotional intelligence outcomes, these changes should not be attributed exclusively to AI tools. The reflective design of the intervention, sustained mentor guidance, peer collaboration, and emotionally supportive learning environment all likely contributed to the observed outcomes. Therefore, the improvements are best interpreted as the result of an integrated mentoring ecosystem rather than the isolated effect of AI.

### 6.3 Combined effect on self-efficacy and emotional intelligence

Returning to the first research question, both the qualitative and quantitative findings converge to show that the AI-supported e-mentoring model produced a combined, mutually reinforcing effect on self-efficacy and emotional intelligence, rather than developing each construct independently. This pattern is directly reflected in participants’ own accounts (Qualitative Theme 4: Development of Self-Efficacy and Emotional Intelligence), where increased teaching confidence and reduced anxiety were consistently described alongside greater emotional awareness and calm under classroom pressure. The quantitative results point to a parallel reciprocal relationship between the two constructs: increased teaching confidence enabled participants to manage classroom stress more effectively and respond constructively to challenges, while improved emotional awareness supported persistence, adaptability, and professional resilience in the classroom. This reciprocal pattern is consistent with previous research linking teachers’ emotional competence with instructional effectiveness and well-being (Arteaga-Cedeño et al., 2025; Braun and Hooper, 2024; Eren et al., 2025).

This combined effect appears to result from the shared mechanisms underlying both constructs within the model: authentic teaching experience, collaborative peer interaction, structured human mentoring, and AI-assisted feedback operated together within a single, continuous cycle of practice, reflection, and feedback, rather than as separate tracks for cognitive and emotional development. As participants gained mastery experiences and confidence in their teaching (self-efficacy), they simultaneously developed the emotional resources to sustain that confidence under classroom pressure (emotional intelligence), and vice versa.

Taken together, the qualitative and quantitative findings jointly answer the first research question by showing that the AI-supported e-mentoring model’s effectiveness lies precisely in this integration: it did not simply add an AI tool to an existing mentoring structure, but combined structured mentoring, collaborative digital interaction, reflective practice, and AI-assisted support into a single ecosystem capable of developing self-efficacy and emotional intelligence together, as interdependent components of teacher readiness.

## 7 Conclusion

The current study examined the effectiveness of an AI-supported e-mentoring model in enhancing EFL pre-service teachers’ self-efficacy and emotional intelligence during their practicum experience in the Egyptian context. The quantitative findings revealed statistically significant improvements in both constructs for the experimental group compared to the control group, as well as substantial pre-post gains within the experimental group, with large effect sizes across all analyses. These results indicate that the intervention had a strong and meaningful impact on participants’ professional and emotional development.

The qualitative findings supported and extended these results by showing clear developmental shifts in participants’ teaching practices and emotional responses. Over the course of the intervention, pre-service teachers demonstrated increased confidence in managing classrooms, greater engagement in student-centered instructional strategies, and improved ability to regulate emotions and respond constructively to classroom challenges. These changes reflected a broader transition from teacher-centered and control-oriented practices to more flexible, reflective, and learner-centered approaches.

Overall, the findings suggest that the AI-supported e-mentoring model was effective in bridging the gap between theoretical preparation and real classroom practice by providing continuous opportunities for reflection, collaboration, and guided feedback. The integration of human mentoring, peer interaction, digital platforms, and AI-assisted support created a coherent developmental environment that strengthened both instructional competence and emotional readiness. In conclusion, the study provides evidence that well-structured AI-supported e-mentoring can serve as a powerful approach in teacher education to support the holistic development of EFL pre-service teachers, particularly in contexts where practicum support is limited or inconsistently delivered.

### 7.1 Implications of the study

The findings of this study have important implications for teacher education and the integration of AI in professional development. The demonstrated effectiveness of the AI-supported e-mentoring model in enhancing EFL pre-service teachers’ self-efficacy and emotional intelligence highlights the value of combining structured mentoring, collaborative digital platforms, and AI-driven feedback within practicum settings. Teacher educators can leverage such models to provide continuous, context-sensitive support, foster reflective practice, and bridge the theory-practice gap. Additionally, AI tools can facilitate personalized guidance, promote adaptive instructional strategies, and strengthen emotional and social competencies, which are critical for effective classroom management and student engagement. Policymakers and program designers may consider incorporating AI-supported mentoring as a scalable, evidence-based approach to enhance pre-service teacher readiness, resilience, and motivation, ultimately contributing to higher-quality teaching and more positive learning outcomes in diverse educational contexts.

### 7.2 Limitations and directions for future research

Despite the promising outcomes of the AI-supported e-mentoring model, several limitations should be acknowledged. First, the study involved a relatively small sample drawn from a single institution, which may limit the generalizability of the findings to other educational contexts. This limited sample size also did not permit a robust examination of the structural validity of the study instruments (e.g., via confirmatory factor analysis), and future research with larger samples should verify whether the underlying factor structures hold in similar populations. As noted earlier, a sensitivity analysis indicated that the study was adequately powered to detect only large effects, meaning smaller but potentially meaningful effects may have gone undetected; future studies should employ larger and more diverse samples across multiple institutions.

Second, all participants were female pre-service teachers, which limits gender representation and the generalizability of the findings across different practicum contexts. This sampling characteristic was influenced by the institutional practicum structure, where pre-service teachers are placed in gender-segregated schools and female student teachers are assigned to girls’ schools. Since the intervention required school visits, classroom observation, mentoring, and continuous follow-up, access to male practicum settings was constrained by administrative regulations and cultural considerations. Furthermore, female students constituted the majority of the English language cohort (approximately 80%), which also influenced sample composition. Future studies are encouraged to include male pre-service teachers and examine whether AI-supported e-mentoring produces comparable outcomes across gender groups and different practicum settings.

Third, since random allocation was conducted at the school level, observations from pre-service teachers within the same school are not fully independent, as they may share contextual influences beyond the intervention itself. This clustering was not modeled statistically, and future studies with more schools should apply multilevel modeling to address it. Fourth, the intervention was conducted within a single practicum semester, which restricts the ability to examine long-term sustainability of the observed improvements in self-efficacy and emotional intelligence. Longer longitudinal designs are therefore recommended to investigate whether these gains persist beyond the practicum period.

Fifth, some participants were initially hesitant to record or document classroom activities, particularly during the early stages of the intervention, which slightly limited the richness of observational and reflective data. Future research may consider stronger ethical reassurance procedures or alternative data collection strategies to address this issue. Sixth, the study focused on second-year pre-service teachers at the preparatory stage of their practicum, rather than more advanced or in-service teachers, which may limit applicability to other stages of teacher development. Finally, the relatively short duration of the intervention further limits conclusions regarding long-term developmental effects.

Future research could address these limitations in several ways. Expanding the study to include a broader range of institutions, including rural and male-dominated contexts, would enhance generalizability. Longitudinal studies could examine whether improvements in self-efficacy and emotional intelligence are sustained and transferred into actual teaching practice after graduation. In addition, integrating AI-supported e-mentoring across multiple stages of teacher education may provide insights into developmental trajectories and optimal scaffolding strategies. Future work may also explore more advanced AI applications, such as virtual teaching simulations or AI-driven classroom analytics, to further support reflective practice, instructional decision-making, and classroom management. Finally, examining how cultural context, institutional constraints, and gender dynamics interact with AI-supported professional development could deepen understanding of how such interventions operate in different educational environments.

## Data Availability

The raw data supporting the conclusions of this article will be made available by the authors, without undue reservation.
