# Supplementary material for: An AI-supported E-mentoring model to develop EFL pre-service teachers’ self-efficacy and emotional intelligence
Source: Front Psychol. 2026 Sep 7;17:1853510. doi: 10.3389/fpsyg.2026.1853510 (PMC13591767; doi:10.3389/fpsyg.2026.1853510)
Supplement: Supplementary file 1 [file Data_sheet_1.pdf]

Supplementary Material

Appendix A

Pre-Intervention Semi-Structured Interview Questions

A. Classroom Management and Student Engagement

- What does classroom management mean to you?
- On a scale from 1 to 10, how would you rate your classroom management skills?
- What strategies do you think are most effective for managing a classroom?
- How would you handle a sleepy student during a lesson?
- How would you manage students who are troublemakers, talkative, demotivated, or showy?
- What aspects of classroom management do you feel you need further training in?

B. Instructional Practices

- What types of teaching activities and techniques do you currently use in your practicum?
- How confident are you in your ability to perform communicative teaching?
- Why do you feel confident or not confident in your ability to perform communicative teaching?
- Which teaching skills do you find most challenging (reading, writing, speaking, listening, grammar, vocabulary) and why?
- What additional training do you feel you need in teaching practices before starting your practicum?

Appendix B

Post-Intervention Semi-Structured Interview Questions

- How has your understanding of classroom management changed after the intervention?
- On a scale from 1 to 10, how would you now rate your classroom management skills?
- What strategies do you now consider most effective for managing a classroom?
- How would you handle a sleepy student during a lesson, after the training?
- How would you manage students who are troublemakers, talkative, demotivated, or showy?
- Which teaching activities and techniques do you plan to use most frequently in the coming term?
- How confident do you feel now about your ability to perform communicative teaching? Please explain why.
- Which teaching skill do you currently find most challenging (grammar, vocabulary, reading, writing, listening, speaking) and why?
- Do you think the training you received was beneficial? Please explain how and why.

Appendix C

Teacher's Sense of Efficacy Scale (TSES, Adapted)

Instructions:

- This questionnaire is designed for understanding the difficulties pre-service teachers face during their practicum
- There is no right or wrong answer; please indicate your opinion about each of the statements below. Your answers are confidential.

A. Efficacy in Classroom Management

Item	1 = Nothing	2 = Very Little	3 = Some Influence	4 = Quite a Bit	5 = A Great Deal
1. How well can you control disruptive behavior in the classroom?					
2. How clearly can you communicate your expectations about student behavior?					
3. How effectively can you establish routines to keep activities running smoothly?					
4. How well can you get students to follow classroom rules?					
5. How effectively can you calm a disruptive or noisy student?					
6. How well can you manage each group of students with a consistent classroom system?					
7. How effectively can you prevent a few problem students from disrupting the entire lesson?					
8. How effectively can you respond to defiant students?					

B. Efficacy in Student Engagement

Item	1 = Nothing	2 = Very Little	3 = Some Influence	4 = Quite a Bit	5 = A Great Deal
1. How well can you reach the most difficult students?					
2. How effectively can you help students think critically?					
3. How well can you motivate students who show low interest in schoolwork?					
4. How effectively can you help students believe they can succeed in schoolwork?					
5. How well can you encourage students to value learning?					
6. How effectively can you foster student creativity?					
7. How well can you help students who are failing improve their understanding?					
8. How effectively can you involve families in supporting student learning?					

C. Efficacy in Instructional Practices

Item	1 = Nothing	2 = Very Little	3 = Some Influence	4 = Quite a Bit	5 = A Great Deal
1. How well can you respond to difficult questions from your students?					
2. How effectively can you gauge student comprehension of your lessons?					
3. How well can you design meaningful questions for your students?					
4. How effectively can you adjust lessons to match students’ individual levels?					
5. How well can you implement a variety of assessment strategies?					

Item	1 = Nothing	2 = Very Little	3 = Some Influence	4 = Quite a Bit	5 = A Great Deal
6. How effectively can you provide alternative explanations or examples when students are confused?					
7. How well can you apply alternative teaching strategies in your classroom?					
8. How effectively can you challenge very capable students with appropriate tasks?					

Source: Tschannen-Moran, M., & Woolfolk Hoy, A. (2001). Teacher efficacy: Capturing an elusive construct. Teaching and Teacher Education, 17, 783-805. https://www.sciencedirect.com/science/article/abs/pii/S0742051X01000361

Appendix D

Trait Emotional Intelligence Questionnaire (TEIQue)

Instructions:
Please answer each statement below by circling the number that best reflects your degree of agreement or disagreement. Work quickly and answer as accurately as possible. There are no right or wrong answers. Responses range from 1 = Completely Disagree to 7 = Completely Agree.

Item No	Statement	1	2	3	4	5	6	7
1	Expressing my emotions with words is not a problem for me.	○	○	○	○	○	○	○
2	I often find it difficult to see things from another person’s viewpoint.	○	○	○	○	○	○	○
3	On the whole, I’m a highly motivated person.	○	○	○	○	○	○	○
4	I usually find it difficult to regulate my emotions.	○	○	○	○	○	○	○
5	I generally don’t find life enjoyable.	○	○	○	○	○	○	○
6	I can deal effectively with people.	○	○	○	○	○	○	○
7	I tend to change my mind frequently.	○	○	○	○	○	○	○
8	Many times, I can’t figure out what emotion I'm feeling.	○	○	○	○	○	○	○
9	I feel that I have a number of good qualities.	○	○	○	○	○	○	○
10	I often find it difficult to stand up for my rights.	○	○	○	○	○	○	○
11	I’m usually able to influence the way other people feel.	○	○	○	○	○	○	○
12	On the whole, I have a gloomy perspective on most things.	○	○	○	○	○	○	○
13	Those close to me often complain that I don’t treat them right.	○	○	○	○	○	○	○
14	I often find it difficult to adjust my life according to the circumstances.	○	○	○	○	○	○	○
15	On the whole, I’m able to deal with stress.	○	○	○	○	○	○	○
16	I often find it difficult to show my affection to those close to me.	○	○	○	○	○	○	○

Item No	Statement	1	2	3	4	5	6	7
17	I'm normally able to "get into someone's shoes" and experience their emotions.	○	○	○	○	○	○	○
18	I normally find it difficult to keep myself motivated.	○	○	○	○	○	○	○
19	I'm usually able to find ways to control my emotions when I want to.	○	○	○	○	○	○	○
20	On the whole, I'm pleased with my life.	○	○	○	○	○	○	○
21	I would describe myself as a good negotiator.	○	○	○	○	○	○	○
22	I tend to get involved in things I later wish I could get out of.	○	○	○	○	○	○	○
23	I often pause and think about my feelings.	○	○	○	○	○	○	○
24	I believe I'm full of personal strengths.	○	○	○	○	○	○	○
25	I tend to "back down" even if I know I'm right.	○	○	○	○	○	○	○
26	I don't seem to have any power at all over other people's feelings.	○	○	○	○	○	○	○
27	I generally believe that things will work out fine in my life.	○	○	○	○	○	○	○
28	I find it difficult to bond well even with those close to me.	○	○	○	○	○	○	○
29	Generally, I'm able to adapt to new environments.	○	○	○	○	○	○	○
30	Others admire me for being relaxed.	○	○	○	○	○	○	○

Source: Petrides, K. (2009). *Psychometric Properties of the Trait Emotional Intelligence Questionnaire*. In C. Stough, *Assessing Emotional Intelligence* (pp. 85–99). Springer. https://link.springer.com/chapter/10.1007/978-0-387-88370-0_5
